# *Bifidobacterium animalis* Subspecies *lactis* CECT 8145 Affects Markers of Metabolic Health in Dogs During Weight Gain and Weight Loss

**DOI:** 10.3390/ani16020259

**Published:** 2026-01-15

**Authors:** Sarah M. Dickerson, Claire L. Timlin, Fiona B. Mccracken, Patrick Skaggs, Sophie L. Nixon, Richard Day, Craig N. Coon

**Affiliations:** 1 Four Rivers Kennel LLC, Walker, MO 64790, USA; 2 ADM R&D Health & Wellness, 3rd Floor, The Minster Building, 21 Mincing Lane, London EC3R 7AG, UK

**Keywords:** metabolic health, postbiotic, probiotic, canine weight gain, obesity, postprandial glucose, *Bifidobacterium animalis*

## Abstract

This study explored the effects of *Bifidobacterium animalis* subspecies *lactis* CECT 8145 (*B. animalis* CECT 8145)—in both live probiotic and heat-treated postbiotic form—on metabolic health and digestion in male and female Labrador Retrievers during weight gain and loss. The study consisted of two, seven-week phases: weight gain (200% maintenance energy intake; Phase (1) and weight loss (100% maintenance energy requirement for ideal weight; Phase (2), separated by a 2-week washout period. In each phase, forty-five adult Labrador Retrievers (1.6–12.5 years) were randomly assigned to daily supplementation with live *B. animalis* CECT 8145 probiotic (PRO, *n* = 15), heat-treated *B. animalis* CECT 8145 postbiotic (POST, *n* = 15), or placebo control (CON, *n* = 15). Body weight, body condition score, fecal quality and food consumption were monitored throughout the study, and body composition, fecal, and blood samples were analyzed at the beginning and end of each phase. Digestibility was evaluated at the end of each phase. Post-prandial glucose responses were affected by intervention during weight loss, with a 6% reduction in the area under the curve (AUC) in POST compared to CON dogs (*p* = 0.035). Glucagon was decreased in females supplemented with POST (*p* = 0.0014), while POST males showed increased glucagon-like peptide-1 (GLP-1) compared to CON (*p* = 0.016) during weight gain. Serum GGT levels decreased, within the normal reference range, in POST compared to CON dogs during weight gain (post hoc *p* = 0.041). Fecal isovalerate was also reduced and fat digestibility increased (*p* = 0.026) in POST compared to CON (*p* = 0.018) during weight gain. There was a significant association between the group and gastric inhibitory polypeptide (GIP), with a decrease in GIP in POST over time (*p* = 0.030), and glucagon tended to be decreased in POST compared to CON (*p* = 0.073). Overall, these findings suggest supplementation with postbiotic *B. animalis* CECT 8145 may improve certain markers of Labrador retrievers’ metabolic health.

## Simple Summary

Obesity is a key risk factor for metabolic disorders and chronic inflammation. With 59% of pet dogs in the USA recently estimated to be overweight or obese, it is crucial to investigate strategies to proactively support metabolic health in dogs, and postbiotics may play a role in this. This study explored how supplementation with *Bifidobacterium animalis* subspecies *lactis* CECT 8145 affects metabolic health markers in Labrador Retriever dogs during weight gain and loss. The study had two phases: a weight-gain phase where dogs were overfed, and a weight-loss phase where overweight dogs were fed just enough to maintain their ideal weight. During each phase, forty-five adult Labradors were divided into three groups: one received the live bacterial supplement (probiotic), another received the heat-treated version (postbiotic), and the third group received a placebo. Dogs’ weight, body fat, fecal biomarkers, and overall health were monitored throughout the study. During weight gain, both probiotic and postbiotic supplements affected certain fecal and blood biomarkers linked to digestion and metabolism. Most notably, the postbiotic reduced blood sugar after consuming kibble in the weight-loss phase. Overall, the study suggests that supplementing dogs with *Bifidobacterium animalis* subspecies *lactis* CECT 8145 may influence metabolic health markers as seen through changes in blood and fecal markers in Labradors during weight change.

## 1. Introduction

Obesity and related metabolic disorders are growing concerns in companion animals, particularly in dog breeds such as Labrador Retrievers, which may be genetically predisposed to weight gain [1,2]. The incidence of obesity in dogs mirrors trends in human populations, leading to a range of health issues including insulin resistance, cardiovascular disease, osteoarthritis, and reduced life expectancy [3,4,5,6,7,8]. In the U.S., the prevalence of dogs that are overweight or obese was estimated to be 59% in 2022, according to the U.S. Pet Obesity Report of the Association for Pet Obesity Prevention [9]. Given these challenges, there is a need for effective strategies to manage and mitigate the impact of obesity in dogs.

The gut microbiome has emerged as a critical player in metabolic health, influencing energy balance, nutrient absorption, and inflammatory responses. Probiotics, including those containing beneficial bacterial strains like *Bifidobacterium animalis* (*B. animalis*), have been proposed as potential modulators of the gut microbiome that could improve metabolic outcomes [10,11,12]. While probiotics are widely used in humans to support metabolic health, their application in veterinary practice, particularly concerning canine obesity, remains underexplored.

Previous studies in humans and rodents have demonstrated that *B. animalis* subspecies *lactis* CECT 8145 (*B. animalis* CECT 8145) can enhance gut health, support weight management, and improve metabolic markers [10,11,12]. Probiotic supplementation with this specific strain has demonstrated altered energy and lipid metabolism in Caenorhabditis elegans [13]. *B. animalis* CECT 8145 has also been shown to affect markers of digestive health and the gut microbiome in healthy adult dogs [14]. However, the effects of *B. animalis* supplementation in dogs during metabolic stress, specifically during phases of weight gain and weight loss, have not been thoroughly investigated. Furthermore, there is limited understanding of how different forms of administration—live probiotic versus nonviable postbiotics—might influence these outcomes. The International Scientific Association for Probiotics and Prebiotics (ISAPP) provides the most widely accepted definition of postbiotics as “a preparation of inanimate microorganisms and/or their components that confers a health benefit on the host.” Postbiotic strains may have greater resilience to heat and moisture than their live counterparts, which would allow for maintained functionality across a wider range of processing environments and finished formats. This would be especially important in pet food as these products are often subject to elevated temperatures during production and are not intended for immediate consumption.

This study aimed to address the core question of whether supplementation with *B. animalis* CECT 8145 influences metabolic health markers and digestive function in dogs undergoing metabolic stress, and whether these effects differ between the live probiotic and heat-treated postbiotic forms. Specifically, we evaluated the effects of both formulations in Labrador Retrievers during controlled periods of weight gain and weight loss, a breed and physiological context known to confer increased susceptibility to obesity-related metabolic disturbances. We hypothesized that supplementation with *B. animalis* CECT 8145 would improve markers of metabolic health via modulation of digestive processes, and that probiotic and postbiotic forms would have similar efficacy. By directly comparing live and heat-treated preparations under conditions of positive and negative energy balance, this study seeks to inform future use of microbiome-based dietary interventions for supporting metabolic health in dogs at risk of obesity and contributes to the broader understanding of probiotics and postbiotics in companion animal nutrition.

## 2. Materials and Methods

All experimental procedures were approved by the Institutional Animal Care and Use Committee at Four Rivers Kennel under Protocol FRK-52. This study was divided into two phases: (1) where feed amounts were increased to stimulate weight gain and (2) where subjects were under calorie restriction to induce weight loss. These phases were treated as separate experiments.

### 2.1. Animals and Housing

Forty-five adult working Labrador Retrievers were enrolled in each phase of the study (22 males and 23 females in Phase 1; 20 males and 25 females in Phase 2). For Phase 1, starting body weight and age averaged 29.82 ± 3.68 (SD) kg and 6.27 ± 3.36 (SD) years of age (range 1.6 to 12.3 years), respectively. For Phase 2, starting body weight and age averaged 33.16 ± 4.50 (SD) kg and 8.39 ± 3.05 (SD) years of age (range 2.9 to 12.5 years), respectively. Dogs enrolled in both seven-week phases underwent a 2-week washout period between phases in which they were fed the study diet alone. In order to ensure all subjects were adequately overweight to undergo weight loss in Phase 2, the 18 dogs with the lowest body condition score and body fat percentage at the end of Phase 1 were replaced. All dogs were individually housed in temperature-controlled kennels with access to outdoor socialization yards for 6–8 h per day when weather permitted. There were no changes to the animals’ normal daily activity as a means of inducing weight gain or loss. All animals had free access to automatic waterers in both the kennels and the yards. All animals were up to date on vaccinations and received monthly prophylactic heartworm and parasite prevention. Body weights and body condition scores (BCSs) were measured weekly on each animal.

### 2.2. Diets and Experimental Intervention

Dogs were maintained on the standard kennel diet (MFA Gold-N Pro, MFA Incorporated, Columbia, MO, USA) and fed once per day in the morning for both phases of the study. At the start of each phase, dogs were blocked by sex, age, BCS, and body weight and randomly assigned to one of three groups, receiving either:

(1) a capsule containing maltodextrin placebo control (CON).
(2) a capsule containing 5 billion colony forming units (CFU) of live *Bifidobacterium animalis* subsp. *lactis* CECT 8145 probiotic (PRO), or
(3) a capsule containing 5 billion cells of heat-treated *Bifidobacterium animalis* subsp. *lactis* CECT 8145 postbiotic (Priome^®^ MH; POST), equivalent to 17 mg of postbiotic when prepared from a batch at a concentration of 3 × 10^11^ cells/gram.

Capsules had a fill weight of 330 mg. Supplements were administered once daily in a Greenies Pill Pocket with the dog’s meal. Flavors varied throughout the study, but all dogs received the same flavor on any given day. Researchers were blinded to supplement assignments until the completion of the study and statistical analyses. In Phase 1, dogs were fed 200% of their maintenance energy requirements to stimulate weight gain. In Phase 2, dogs were fed 100% of their maintenance energy requirements for their ideal weight, to promote weight loss. Beginning on day 0 of each phase, dogs were given a seven day acclimation period to their new feeding amounts and supplements. In Phase 1, dogs were offered 200% of requirements immediately, but to ease the transition and avoid problems with bloating in Phase 2, food amounts were gradually decreased over the acclimation week. In Phase 1, males were offered on average 1195 ± 49 g of kibble per day, and females were offered 1134 ± 53 g of kibble per day. In Phase 2, males were offered 543 ± 21 g per day, and females were offered 484 ± 21 g per day. The diets were weighed out in grams each day and dogs were provided at least 45 min to consume their portions. Any food not consumed was collected and the weight was recorded. The metal food bowls were washed daily after the meal with warm soapy water, rinsed and then disinfected by briefly dipping bowls in diluted bleach water and set out to dry. Bowls were sanitized monthly using a commercial dishwasher.

### 2.3. Digestibility

Beginning day 42 (last week of each study phase), apparent total tract digestibility was estimated using the indicator method. Dogs received 2 g of titanium dioxide administered orally via gel capsules for five consecutive days. Fecal samples were collected for three days beginning on the 4th day of titanium administration. Aliquots of equal mass from each daily sample were then mixed and homogenized for proximate analysis testing at either Nestlé Purina Analytical Labs (NPAL, St. Louis, MO, USA; Phase 1) or University of Georgia’s Agricultural & Environmental Services Laboratories (College Station, Athens, GA, USA; Phase 2). Analyses were performed at different labs due to discontinuation of the required titanium analysis. Samples were assessed for moisture by vacuum oven, crude protein by combustion, crude fiber, crude fat by acid hydrolysis, ash at 600 °C, and titanium (indicator). All values were converted to a dry matter basis before analysis. Nitrogen-free extract (NFE) was calculated as 100—% crude protein—% crude fiber—% crude fat—% ash. Apparent digestibility coefficient (ADC) was calculated for dry matter, protein, fat, and NFE using the following published equation [15]:

$${Nutrient}_{flow\;}={Nutrient}_{f}\;\times \;\frac{{{TiO}_{2}}_{i}}{{TiO_{2}}_{f}}$$

$$ADC\;\left(\%\right)=\frac{{Nutrient}_{i}-{Nutrient}_{flow}}{{Nutrient}_{i}}\times 100\%$$

where *Nutrient_flow_* is measured g/d, *Nutrient_f_* represents nutrient content of the feces in g/kg, *TiO*_2*i*_ represents the consumed TiO_2_ in g, *TiO*_2*f*_ represents the TiO_2_ content of the feces in g, and *Nutrient_i_* is the nutrient intake in g/d.

### 2.4. Fecal Quality Scoring

Fecal quality scores were recorded each day for the duration of the trial. Study technicians were trained and evaluated for proper stool scoring before beginning the trial to ensure reliability. Fecal consistency was scored based on what each dog had produced overnight in individual kennels. If a dog had not produced a fecal sample overnight, the dog was accompanied outside individually to obtain a stool score. Scoring was performed as follows: 1—liquid diarrhea, no form, could be poured; 2—loose diarrhea, no form, will take the shape of a container; 3—very moist, soft, and partially formed; 4—firm, well-formed, easy to pick up and leaves no marks on floor; 5—little to no moisture, hard and crumbled easily.

### 2.5. Body Composition

Body composition was assessed using a GE Lunar Prodigy dual-energy X-ray absorptiometry (DEXA) scanner (GE HealthCare, Chicago, IL, USA). This occurred on days 7 and 8 and 35 and 36 in Phase 1, then on days 7 and 8 and 49 and 50 in Phase 2. Dogs were sedated with dexmedetomidine hydrochloride (62.5 mcg/m^2^; Dexdomitor, Zoetis, NJ, USA) and butorphanol tartrate (0.05 mg/lb., Zoetis, NJ, USA) by a licensed veterinarian for the procedure. Additional isoflurane gas with oxygen was administered if necessary. Dogs were aligned on the scanner in ventral recumbency. Bone mineral density, fat mass in grams, lean mass in grams, fat percentage, and total tissue mass in grams were quantified with the scanner’s computer software. Lean: fat ratio was also calculated by dividing lean mass in grams by fat mass in grams.

### 2.6. Post-Prandial Glucose Assessment

In weeks 2 and 7 of each phase, post-prandial glucose levels were measured using the iPet Pro glucose monitoring system (Ultimed Incorporated, Excelsior, MN, USA). Blood glucose was measured prior to eating and 15, 30, 45, 60, 90, 120, and 180 min after the meal was provided by pricking the pinna of the ear with a 28 g lancet (EverPaw, Oakland, CA, USA) in a spring-loaded, vacuuming lancing device (Genteel LLC, Tualatin, OR, USA). Monitors and strips were evaluated each day using the provided control solution to ensure the accuracy of readings was within acceptable range. In Phase 1, dogs were fed their full meal, but there was a wide variation in the amount of kibble consumed and how long it took to consume food, with some dogs taking up to 15 min. To prevent this in Phase 2, only a 350 g portion of the dog’s meal was provided. In Phase 2, all meals were consumed within 5 min. Any remaining daily allotment was provided at the conclusion of the glucose test.

### 2.7. Sample Collection and Analysis

Fasted blood samples were collected by a trained veterinary technician via jugular venipuncture into vacutainer tubes at the beginning (day 7 Phase 1; day 10 Phase 2) and end (day 45 Phase 1; day 49 Phase 2) of each phase. One 8.5 mL volume of blood was collected into vacutainers with clot activator and serum separator gel. Blood for serum collection was clotted at room temperature for at least 30 min, centrifuged at 1500× *g* for 15 min, then separated into aliquots. Two serum aliquots were stored at 4 °C for analysis of blood chemistry panels, liver panel, and triglycerides. Chemistry and liver panels were analyzed in-house using a VetScan VS2 (Abaxis/Zoetis, Union City, CA, USA) chemistry analyzer and processed within 48 h. Serum triglycerides were analyzed by Mizzou Vet Diagnostic Lab (Columbia, MO, USA). Remaining serum was stored at −80 °C. A second 2 mL volume of blood was collected into vacutainers containing K_2_EDTA for complete blood counts and gut hormone analysis. Complete blood counts were assessed within an hour of collection using a VetScan HM5 (Abaxis/Zoetis, USA). An aliquot of whole blood (1 mL) used for gut hormone analysis was mixed with a protease inhibitor cocktail, DPP IV inhibitor, and Pefabloc SC (MilliporeSigma, Burlington, MA, USA), centrifuged at 1000× *g* for 10 min, and plasma was aliquoted and frozen at −80 °C until analysis. Amylin (total), active ghrelin, gastric inhibitory polypeptide (GIP), glucagon-like peptide-1 (GLP-1), glucagon, insulin, leptin, pancreatic polypeptide (PP), and peptide YY (PYY) were quantified using the MILLIPLEX Canine Gut Hormone Magnetic Bead Panel (CGTMAG98K, Millipore-Sigma, Burlington, MA, USA) on MAGPIX (Luminex, Austin, TX, USA) following manufacturer’s instructions.

Fecal samples were collected on days 9 and 39 of each phase of the study. Fecal collections were performed before digestibility analysis to prevent any potential confounding effects of the titanium indicator. Two 1 g samples were collected and sent to Texas A&M Veterinary Medical Diagnostic Lab (College Station, Athens, TX, USA) for analysis of IgA and short-chain fatty acids (SCFA). Fecal extract was collected for analysis of fecal pH and fecal ammonia. A 1-g sample was weighed out and diluted with 5 mL of distilled water, vortexed for 30 s, then the pH was measured using a calibrated pH meter. Tubes were centrifuged at 2500× *g* for 20 min and supernatant was collected and stored at −80 °C until ammonia analysis using a rapid ammonia assay kit (K-AMIAR, Neogen, Lansing, MI, USA). Samples for ammonia were further diluted in distilled water for a total dilution of 1:40. Lastly, collected samples were analyzed for fecal moisture by loss on drying, performed by drying no more than 5 g in an oven at 133 °C for 4 h.

### 2.8. Statistical Analysis

Statistical analysis was performed in SAS Studio 3.8 (SAS Institute, Cary, NC, USA). Food consumption data were analyzed as a proportion of food consumed to food offered and in total grams consumed. For both food consumption and stool quality data, measurements and scores were averaged by week for statistical analysis. If necessary, data were given a Box-Cox transformation to assume the normality of residuals assumption. Digestibility results were analyzed among experimental groups using a one-way ANOVA. All other measurements were analyzed using a repeated measures mixed model with dog within group as the repeated subject. Blood glucose levels during the post-prandial glucose curve were analyzed using all timepoints across both assessments, fixed effects of time, group, and group*time interaction, and sex included as a random effect. All other models contained fixed effects of sex, time, group, group*time, and sex*group interactions. Non-significant sex*group interactions were removed from the model. Significant effects in all models were followed up with Tukey’s post hoc analysis. Any models with significant sex*group interactions were re-analyzed with female and male data separated. All repeated measures models had covariance structure selected based on variance and covariances of the unstructured model and the lowest corrected Akaike information criterion (AICc). Significance was set at *p* ≤ 0.05 and tendencies were set at 0.05 < *p* ≤ 0.10. As all measurements (week 2 and week 7) were taken during supplementation, both group and group*time interaction were interpreted to be associated with the intervention group. Data is presented as least square means with their standard errors (SEM). Any transformed data was reported as the back-transformed means and estimated SEM according to Jorgensen and Pedersen [16].

## 3. Results

### 3.1. Food Consumption

**Figure 1:**
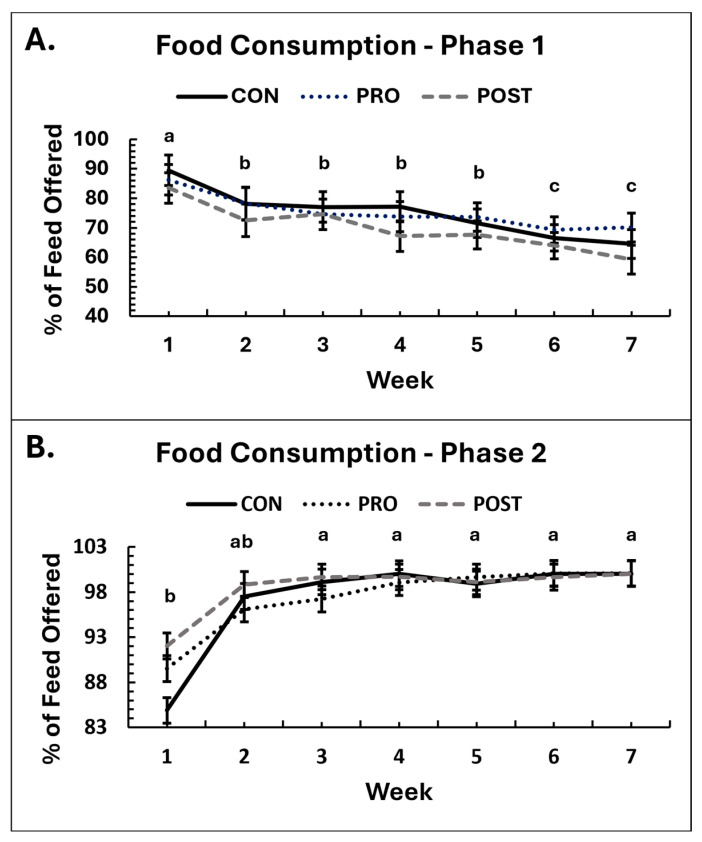
Average weekly food consumption, displayed as percentage of food consumed over food offered, for groups receiving placebo control (CON), *B. animalis* subsp. *lactis* CECT 8145 probiotic (PRO) or heat-treated postbiotic (POST) throughout the duration of the study ((**A**): Phase 1, (**B**): Phase 2). Means sharing the same superscript letter are not significantly different; means with different superscripts differ significantly between weeks (*p* < 0.05).

During Phase 1, there was no difference among groups in the amount of food consumed (*p* ≥ 0.14), although the consumption based on percentage of food offered tended to differ over time, with dogs in POST consuming the least (Supplementary Table S1; *p* = 0.0925) but no significant differences between groups were identified by post hoc analysis (*p* ≥ 0.13). Over time, food consumption voluntarily decreased in all groups during Phase 1 (Figure 1; *p* < 0.001) even though dogs were consistently offered the same amount of food daily. Despite this reduction, by week 7 dogs were still consuming 170% of their maintenance energy requirements for their ideal weight, indicating continued intake of a calorie excess. Males consumed more food in grams than females (*p* = 0.0091), as can be expected due to their larger body weights and greater caloric requirement, but the percentage consumption did not differ between sexes.

In Phase 2, there was no significant effect of treatment group on food intake (*p* ≥ 0.15). Although a group-by-time interaction was observed for the percentage of food consumed (*p* = 0.035), this was due to changes between week 1 and week 2 rather than a true effect of supplementation. The percentage consumption data were skewed, as most dogs ate all their food after the acclimation period. Correcting the data distribution using transformations was not possible, so these results should be interpreted with caution. When data from the acclimation period were excluded, no significant group differences were observed (*p* = 0.65). Food consumption increased over time (*p* < 0.001) and this effect persisted (*p* < 0.001), when data were analyzed without the acclimation week. As in Phase 1, males consumed more food than females (*p* = 0.026).

### 3.2. Body Weights, Condition, and Composition

**Figure 2:**
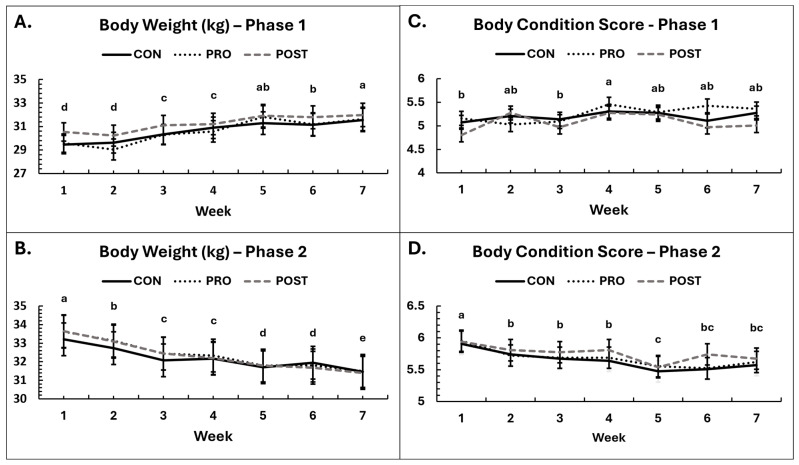
Body weight and body condition scores. Average body weight in kilograms by experimental group throughout the study in Phase 1 (**A**) and Phase 2 (**B**). Body condition scores by experimental group in Phase 1 (**C**) and Phase 2 (**D**). Condition scores are based on a scale of 1 to 9, with 1 extremely thin and 9 very obese. Ideal score range: 4 to 5. Means sharing the same superscript letter are not significantly different; means with different superscripts differ significantly between weeks (*p* < 0.05).

There were significant changes over time in body weight, condition and composition in each phase as was anticipated by study design. In Phase 1, body weights increased (*p* < 0.001, Figure 2), and, on average, dogs gained 1.86 kg, or about 6.24% of starting body weight from the initiation of the study. In Phase 2, weights steadily decreased over time (*p* < 0.001, Figure 2), and dogs lost 2.04 kg, or about 6.08% of starting body weight. However, there were no differences in bodyweight change among groups in either Phase 1 (*p* ≥ 0.13) or Phase 2 (*p* ≥ 0.71). In both phases, males weighed significantly more than females, as expected (Phase 1: 32.82 ± 0.67 kg vs. 28.82 ± 0.65 kg, *p* < 0.001; Phase 2: 35.30 ± 0.75 kg vs. 29.29 ± 0.67 kg, *p* < 0.001). Body weights are summarized in Figure 2. No group × sex interactions were observed for either phase (*p* ≥ 0.73).

Similar to body weights, BCS did not differ among groups in Phase 1 (*p* ≥ 0.26) or Phase 2 (*p* ≥ 0.83). Changes in BCS occurred over time in each phase, aligning with the weight data. In Phase 1, BCS increased slightly over time in all three groups (*p* = 0.0083, Figure 2) but plateaued by the last 3 weeks. In Phase 2, BCS decreased over time in all three groups (*p* < 0.001, Figure 2). In Phase 1, BCS tended to be greater in males than in females (*p* = 0.070), but no such differences were detected in Phase 2 (*p* = 0.23).

Body composition changed in each phase (Supplementary Table S2), as anticipated with this study design. In Phase 1, total tissue mass (*p* < 0.001), fat mass in grams (*p* < 0.001), and body fat percentage (*p* < 0.001) all increased over time. Dogs increased their body fat percentage by an average of 4.76% and gained on average 1.68 kg of fat tissue. Lean tissue mass decreased (*p* < 0.001) on average 0.42 kg while dogs gained fat. As a result, the lean: fat mass ratio also decreased from 5.95 to 4.37 (*p* < 0.001). There were no differences among groups for bone mineral density (*p* ≥ 0.50), body fat percentage (*p* ≥ 0.63), fat mass (*p* ≥ 0.89), total tissue mass (*p* ≥ 0.82), or lean mass: fat mass ratio (*p* ≥ 0.68). There was a tendency for lean mass to be affected by group (*p* = 0.098) with the decrease in lean mass smallest for the POST group, however there were not pairwise differences between groups by post hoc analysis.

In Phase 2, we observed decreases in total tissue mass (*p* < 0.001), fat mass in grams (*p* < 0.001), and body fat percentage (*p* < 0.001) over time. Dogs lost on average 3.15% body fat and 1.39 kg of fat mass. Lean mass was not affected over time in Phase 2 (*p* = 0.29). The decrease in body fat increased the lean: fat ratio from 2.48 to 3.31 (*p* = 0.0075). Similarly to Phase 1, there were no differences in body composition among groups [body fat percentage (*p* ≥ 0.63), fat mass (*p* ≥ 0.88), lean mass (*p* ≥ 0.27), total tissue mass (*p* ≥ 0.27), lean mass: fat mass ratio (*p* ≥ 0.79), bone mineral density (*p* ≥ 0.12)].

In both phases, males had greater bone mineral density compared to females (Supplementary Table S2, Phase 1: 0.66 ± 0.01 vs. 0.61 ± 0.01, *p* < 0.001; Phase 2: 0.68 ± 0.01 vs. 0.61 ± 0.01, *p* < 0.001). Males also had significantly more lean mass (Phase 1: 24,413 ± 594.04 g vs. 20,412 ± 580.96 g, *p* < 0.001; Phase 2: 23,151 ± 625.98 g vs. 18,577 ± 559.77 g, *p* < 0.001) and total tissue mass (Phase 1: 30,117 ± 624.66 g vs. 26,404 ± 611.80 g, *p* < 0.001; Phase 2: 32,742 ± 701.81 g vs. 26,829 ± 630.01 g, *p* < 0.001) as can be expected. There were no differences in percentage body fat, total fat mass, or lean: fat ratio between sexes in Phase 1 (*p* ≥ 0.24) or Phase 2 (*p* ≥ 0.15). The observed tendency for males to have increased BCS in Phase 1 does not align with the DEXA results and may reflect the scoring technicians mistaking lean muscle mass for fat mass on some dogs. Body condition scoring is a subjective scoring method and as a result, likely to be less accurate than a quantitative method like DEXA.

### 3.3. Post-Prandial Glucose Test

**Figure 3:**
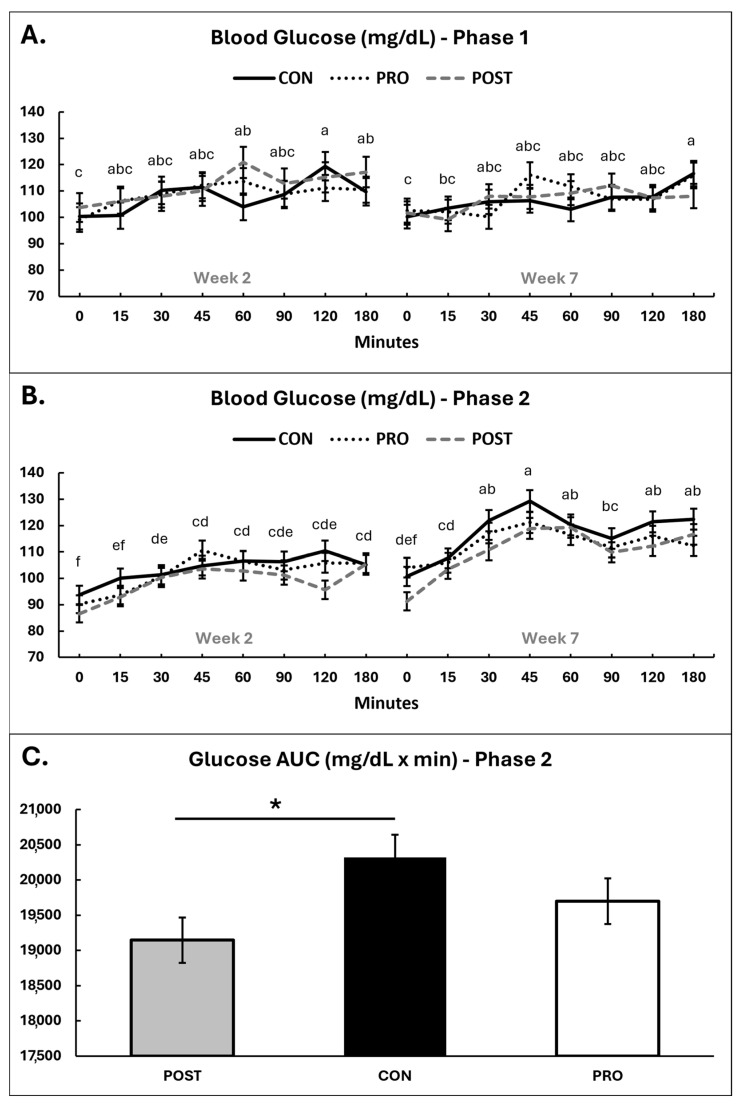
Post-prandial blood glucose for all experimental groups throughout the study in Phase 1 (**A**) and Phase 2 (**B**) and glucose AUC for week 7, Phase 2 (**C**). Time 0 represents baseline glucose levels before the meal. Means sharing the same superscript letter are not significantly different; means with different superscripts differ significantly between weeks (*p* < 0.05). * = *p* < 0.05.

Post-prandial glucose responses are summarized in Figure 3, with individual datapoints plotted in Supplementary Figure S2.

In Phase 1, across all groups blood glucose levels significantly increased post-prandially, as expected (*p* < 0.001), with concentrations rising above baseline at 45 min post meal and remaining elevated until the end of the test at 180 min (Figure 3A). There were no effects of group or group*time interaction (*p* ≥ 0.35). Area under the curve (AUC) did not differ by group or time (*p* ≥ 0.43).

Females had a significantly lower AUC overall in Phase 1 (*p* = 0.038), but this did not differ between groups (*p* ≤ 0.15). Baseline glucose levels did not differ between sex when baseline measures were analyzed separately (*p* = 0.11). There were no differences in baseline glucose reading detected by post-hoc analysis in the glucose curves or with isolated baseline measurements analyzed (*p* ≥ 0.43).

In Phase 1, the AUC and glucose results may be confounded by food consumption as dogs were given their entire meal, so consumption amounts and times were quite variable between dogs. As males were fed on average more food, that may explain the increased AUC compared to females. This potential confounder was addressed in Phase 2 where all dogs were given the same 350 g portion of food, which was consumed in about 5 min for all dogs.

In Phase 2, blood glucose became significantly elevated from baseline by 30 min after eating and remained elevated until the end of the test at 180 min (Figure 3B). There was an association between intervention group and postprandial AUC (*p* = 0.045), where POST had a decreased AUC compared to CON by 6% (post hoc *p* = 0.035) but there was no difference between PRO and CON (post hoc *p* ≥ 0.40 (Figure 3C).

There was a tendency for a relationship between intervention group and postprandial glucose overall (*p* = 0.078), with dogs in POST having lower blood glucose than dogs in CON (post hoc *p* = 0.062) but no difference between PRO and CON (post hoc *p* ≥ 0.45).

There was evidence for an overall relationship between timepoint and postprandial blood glucose in Phase 2, with greater AUC at week 7 compared to week 2 (*p* < 0.001), an increased peak glucose reading at 45 min post-prandial in week 7 (123 ± 2.3 vs. 106 ± 2.2 mg/dL; *p* < 0.01), as well as higher baseline readings in week 7 (99 ± 2.0 mg/dL) compared to week 2 (91 ± 2.5 mg/dL; *p* = 0.01).

### 3.4. Gut Hormones

Gut hormone results are summarized in Table 1. A significant group by sex interaction was observed in Phase 1 for GLP-1, glucagon, and leptin; therefore, male and female data for these 3 analytes were analyzed separately.

In Phase 1, GLP-1 was greater in males supplemented with POST compared to CON (post hoc *p* = 0.016) but there was no difference between PRO and CON (post hoc *p* ≥ 0.21). There was an increase in GLP-1 Phase 1 in the model containing data from both sexes (*p* = 0.018), but when broken down by sex the increase was only significant in males (*p* = 0.041). Glucagon levels in females were significantly lower in POST compared with CON (post hoc *p* ≤ 0.0014). There was a significant increase in glucagon over time in males (*p* = 0.0053) but not females. There was a tendency in females for decreased leptin levels in PRO compared to CON (post hoc *p* = 0.072), but there was no difference between POST and CON (post hoc *p ≥* 0.26). As expected, leptin levels increased over time (*p* < 0.001) in parallel to the increase in body fat over time observed by DEXA and BCS (*p* ≤ 0.028). GIP tended to be affected by supplementation, with POST supplementation tending to decrease GIP (post hoc *p* = 0.073), while GIP levels remained stable for CON and PRO (post hoc *p* ≥ 0.70). Pancreatic polypeptide decreased with weight gain (*p* = 0.019) but was not affected by supplementation. There was a sex difference in Peptide YY in Phase 1, with males having greater circulating concentrations (71.32 ± 6.77 vs. 53.09 ± 6.78 pg/mL, *p* = 0.022), but no association between Peptide YY and supplementation.

In Phase 2, some gut hormones were affected by supplementation although the effects differed from Phase 1. There was an association between GIP and group (*p* = 0.03) with GIP for POST tending to be lower than CON by post hoc comparison (*p* ≥ 0.064 Phase 2). There was a tendency for an association between group and GLP-1 (*p* = 0.064), although there were no differences among Tukey’s post hoc comparisons across all groups and timepoints (*p* ≥ 0.32). Sex differences were observed in GLP-1 (*p* = 0.0051) with males having greater circulating levels than females (6.37 ± 0.78 pg/mL vs. 4.12 ± 0.39 pg/mL). Finally, there was a tendency for a relationship between glucagon and group (*p* = 0.073), where glucagon decreased in POST by day 49 (post hoc *p* = 0.006), while the other groups were not significantly different (*p* ≥ 0.89). Circulating PYY (*p =* 0.0037) and leptin (*p* < 0.001) decreased with weight loss. In contrast, amylin (*p* = 0.0036) and insulin (*p* < 0.001) increased. No significant interactions between sex and group were observed for gut hormones in Phase 2 (*p* ≥ 0.104).

### 3.5. CBCs and Chemistries

Results for complete blood counts are shown in Supplementary Table S3, and serum chemistry results are shown in Supplementary Table S4. All values were within the normal ranges, except for serum globulin which was low in Phase 1 and bile acids which were undetectable for the majority of subjects in both phases.

Several hematology parameters changed over time, with Phase 1 and Phase 2 acting inversely to each other. Eosinophils, basophils, red blood cells (RBC), hemoglobin (HGB), hematocrit (HCT), mean corpuscular volume (MCV), RDW coefficient of variation (RDWc), and RDWs all increased over time with weight gain (*p* ≤ 0.026). In contrast, these markers all decreased over the course of Phase 2 as dogs lost weight (*p* ≤ 0.032). Mean corpuscular hemoglobin concentration (*p* < 0.001), mean platelet volume (MPV, *p* = 0.077), and platelet distribution width (PDW) standard deviation (PDWs, *p* = 0.030) decreased during weight gain and increased during weight loss (*p* ≤ 0.024). The rate of change appeared similar between phases. Regarding serum chemistries, AMY (*p* = 0.0012) and CHOL (*p* = 0.0029) both increased during weight gain, while during weight loss they decreased over time (*p* ≤ 0.027). Albumin (*p* < 0.001) and calcium (*p* = 0.025) significantly increased in Phase 1 but only tended to decrease during weight loss (*p* ≤ 0.067). Sodium was the only parameter whose change was not correlated with weight changes; rather, an effect of time whereby a decrease in serum levels was observed from the start of each phase to the end (*p* ≤ 0.0084).

Unique changes occurred during weight gain that were not observed during weight loss. For example, mean corpuscular hemoglobin (MCH) decreased over time (*p* = 0.011) while platelets increased (*p =* 0.0045), and plateletcrit tended to increase (*p* = 0.072) in Phase 1, but none of these parameters were affected in Phase 2 (*p* ≥ 0.13). Alanine transaminase tended to decrease (*p* = 0.055), and glucose significantly decreased (*p* < 0.001) in Phase 1, but no changes were observed in Phase 2 (*p* ≥ 0.68). Total bilirubin also increased in Phase 1 (*p* < 0.001) with no changes in Phase 2 (*p* = 1.00).

In contrast, changes during weight loss in Phase 2 occurred that had not been observed in Phase 1. Over time, PDW coefficient of variation increased (PDWc, *p* < 0.001) in Phase 2. Alkaline phosphatase (*p* < 0.001) and total protein (*p* < 0.001) decreased with weight loss but were not affected by weight gain (*p* ≥ 0.46). Blood urea nitrogen (*p* < 0.001), creatinine (*p* < 0.001), and globulin (*p* = 0.034) all increased in Phase 2 during weight loss but were not affected by weight gain (*p* ≥ 0.12).

In Phase 1, there were no significant differences between groups for any of the complete blood count parameters (*p* ≥ 0.11). There was a tendency for potassium levels to be higher in CON compared to POST (post hoc *p* = 0.071), with PRO intermediate (*p* ≥ 0.24). Potassium remained within the recommended reference range (3.7–5.8 mmol/L). There was also a significant effect of group on GGT, where POST had lower GGT than CON (*p* = 0.041), with no difference between PRO and CON (*p* ≥ 0.28).

In Phase 2, the only group effect on complete blood count parameters was observed for plateletcrit, which tended to be greater in CON than POST (post hoc *p* = 0.064), with no difference between PRO and CON (*p* ≥ 0.40). Plateletcrit remained within the veterinary reference range. Calcium was the only chemistry parameter affected by group, displaying a significant decrease in PRO compared to POST (post hoc *p* = 0.007) and a tendency for decrease between PRO and CON (post hoc *p* = 0.062). Calcium tended to decrease overall in Phase 2 (*p* = 0.065) It is unclear whether this group effect is due to changes in calcium absorption or utilization during weight loss and what those implications are for long-term weight loss. All groups remained within the 8.3–11.8 mg/dL reference range for calcium.

Sex differences were observed in haematology and serum chemistries. In both phases 1 and 2, females had lower levels of amylase (AMY; Phase 1: 615.66 ± 35.39 vs. 742.68 ± 36.19, *p* = 0.016; Phase 2: 558.31 ± 33.36 vs. 770.01 ± 37.31, *p* < 0.001) and blood urea nitrogen (BUN; Phase 1: 15.27 ± 0.81 vs. 18.34 ± 0.83, *p* = 0.012; Phase 2: 12.79 ± 0.56 vs. 15.67 ± 0.63, *p* = 0.0014) compared to males. Females also had elevated total bilirubin (TBIL; Phase 1: 0.28 ± 0.01 vs. 0.27 ± 0.01, *p* = 0.019; Phase 2: 0.36 ± 0.01 vs. 0.32 ± 0.01, *p* < 0.001), gamma-glutamyl transferase (GGT; Phase 1: 4.05 ± 0.20 vs. 3.16 ± 0.20, *p* = 0.0015; Phase 2: 4.06 ± 0.17 vs. 3.37 ± 0.19, *p* = 0.0081), and red blood cell distribution (RDW) standard deviation compared to males (RDWs; Phase 1: 42.25 ± 0.23 vs. 41.10 ± 0.24, *p* < 0.001; Phase 2: 42.66 ± 0.24 vs. 41.87 ± 0.27, *p* = 0.033). In Phase 1, females had significantly elevated cholesterol (CHOL; 229.30 ± 9.53 vs. 196.87 ± 9.75, *p* = 0.022), while in Phase 2 it was a tendency (226.88 ± 8.55 vs. 203.78 ± 9.56, *p* = 0.079). The agreement between both phases indicates inherent physiological differences between sexes in blood chemistry markers.

In Phase 1 only, males had elevated monocytes (0.50 ± 0.03 vs. 0.38 ± 0.03, *p* = 0.0044) and mean corpuscular hemoglobin concentration compared to females (MCHC; 32.90 ± 0.16 vs. 32.18 ± 0.16, *p* = 0.0031) while females had elevated albumin (ALB; 3.87 ± 0.04 vs. 3.70 ± 0.04, *p* = 0.0037) and alkaline phosphatase (ALP; 56.27 ± 4.15 vs. 43.03 ± 3.25, *p* = 0.015) compared to males. In Phase 2 only, females had elevated phosphorus (4.77 ± 0.07 vs. 4.54 ± 0.08, *p* = 0.031), glucose (95.47 ± 1.22 vs. 89.31 ± 1.36, *p* = 0.0012), and sodium (146.74 ± 0.33 vs. 145.28 ± 0.36, *p* = 0.0047) compared to males.

### 3.6. Digestibility

In Phase 1, apparent total tract digestibility coefficients (ADC) for fat digestibility differed among groups (*p* = 0.025), with greater digestibility in POST compared to CON dogs (post hoc *p* = 0.026) but did not differ for PRO (post hoc *p* ≥ 0.11). Crude protein ADC tended to differ (*p* = 0.072) among groups, with post hoc comparisons revealing a tendency for increased digestibility in POST compared to CON (*p* = 0.062) but not PRO compared to CON (*p* ≥ 0.30). Digestibility results are summarized in Table 2. Supplement did not affect digestibility coefficients for dry matter (DM) and NFE (*p* ≥ 0.12).

In Phase 2, crude protein (*p* = 0.075) and NFE (*p* = 0.079) digestibility tended to differ among groups. Crude protein digestibility tended to be lower in POST compared to CON (post hoc *p* = 0.10), but PRO did not differ (post hoc *p* ≥ 0.13). Nitrogen-free extract tended to be lower in PRO compared with CON (post hoc *p* = 0.067), but POST did not differ (post hoc *p* ≥ 0.35). There was a tendency for a sex effect on dry matter digestibility (*p* = 0.077), with males having slightly greater dry matter digestibility (88.56 ± 1.26 vs. 85.50 ± 1.12), but this did not differ among groups (*p* = 0.36).

Caution is advised when comparing results between phases 1 and 2, as the analytical labs used for each phase were different due to the discontinuation of titanium analysis from the initial analytical lab.

### 3.7. Fecal Parameters

There were no differences among groups in fecal quality scores in Phase 1 (*p* ≥ 0.65) or Phase 2 (*p* ≥ 0.28), and fecal scores remained within normal consistency ranges for the colony. In Phase 1, there was a significant and steady increase in score overtime in all groups (*p* < 0.001, Supplementary Figure S1). In Phase 2, fecal scores also increased after the first week but remained consistent afterwards (Supplementary Figure S1).

Fecal marker results are summarized in Supplementary Table S5. Fecal moisture did not differ among groups in either phase (*p* ≥ 0.15). In Phase 1, fecal moisture decreased over time (*p* = 0.041), which matches the observed increase in fecal quality scores towards more firm stools. In Phase 2, there were no changes over time (*p* = 0.79), aligning with the consistent stool quality scores after week 1.

There was a significant relationship between group and fecal pH (*p* = 0.046) in Phase 1. Although there were no significant or trending pairwise comparisons by Tukey’s post-hoc (*p* ≥ 0.32), fecal pH showed a pattern of increasing in dogs receiving POST, while those receiving CON and PRO reduced pH over time. Fecal pH did not differ by sex, group, or over time (*p* ≥ 0.26) in Phase 1. In Phase 2, there were no differences between groups (*p* ≥ 0.56), but pH decreased over time overall (*p* = 0.012). There was also an effect of sex in Phase 2, with females having greater pH than males (6.36 ± 0.05 vs. 6.18 ± 0.05, *p* = 0.015).

Fecal IgA did not differ among groups in Phase 1 (*p* ≥ 0.21) or Phase 2 (*p* ≥ 0.19), but it decreased over time in all groups (*p* ≤ 0.034) in both phases of the study.

Fecal ammonia concentration showed a tendency for an association with group (*p* = 0.073) in Phase 1, although no pairwise differences were detected (post hoc *p* ≥ 0.44). In Phase 2 there were no differences among groups (*p* ≥ 0.21), but overall fecal ammonia concentrations increased over time (*p* < 0.001).

Several associations between group and fecal SCFA were observed in Phase 1 but not Phase 2. Fecal isobutyrate concentration differed by group over time in Phase 1, increasing in both PRO and POST but decreasing in CON (post hoc *p* = 0.061, 0.072, respectively). In Phase 1, isovalerate also differed among groups over time (*p* = 0.025), with isovalerate decreasing over time in CON but increasing in POST (post hoc *p* = 0.018) and PRO (post hoc *p* = 0.092). Acetate was associated with supplementation (*p* = 0.034), but there were no significant differences in the pairwise comparison. Butyrate (*p* = 0.014) and valerate (*p* = 0.028) were affected by supplementation in Phase 1 and propionate (*p* = 0.062) tended to be associated with supplementation, but there were no differences detected by post hoc pairwise comparison (*p* ≥ 0.11). In Phase 2, there was a tendency for group effect on isovalerate (*p* = 0.092, though no differences by post hoc analysis (*p* ≥ 0.13). Propionate concentrations (*p* = 0.062) also tended to be affected by supplementation, where CON tended to be elevated compared to POST (post hoc *p* = 0.081) but PRO did not differ (post hoc *p* ≥ 0.13). Phase 1 Phase 2. Sex did not affect fecal SCFA in Phase 1 (*p* ≥ 0.12) or Phase 2 (*p* ≥ 0.28).

## 4. Discussion

### 4.1. Key Findings

This study investigated the effects of live and heat-treated *Bifidobacterium animalis* CECT 8145 supplementation on metabolic health and fecal parameters in Labrador Retrievers undergoing controlled weight gain and loss. While supplementation did not significantly alter changes in bodyweight or composition, it influenced gut hormone levels and metabolic markers, particularly in Phase 1, and post-prandial glucose in Phase 2. Several notable findings related to food consumption, digestibility, body composition, stool quality, fecal markers, gut hormones, and blood chemistries may provide new insights into the role of probiotics and postbiotics in canine health management. Notably, the postbiotic supplement tended to have greater impact than the probiotic when provided at this dosage. Additionally, the study revealed metabolic and laboratory blood marker changes associated with weight gain and weight loss, which could be useful for researchers and clinicians investigating pet obesity.

Overall, the probiotic demonstrated a greater effect on markers of metabolic health than the postbiotic. Direct comparisons between live and heat-treated micro-organisms remain limited, and it is therefore unclear whether similar differences are observed across other strains. For this strain, pre-clinical evidence indicates modulation of the insulin–IGF-1 signalling pathway via lipoteichoic acid in the bacterial cell wall. It is plausible that the biological activity of this component differs between live and heat-treated forms, potentially due to differences in structural stability or bioavailability following gastrointestinal transit. Further studies examining the stability and persistence of the micro-organism and its bioactive components in both forms under simulated gastrointestinal conditions would help to clarify the mechanisms underlying these observations.

### 4.2. Postprandial Glucose

During weight loss, supplementation attenuated the increase in post-prandial glucose area under the curve (AUC) seen in the control group. This may indicate a role for *B. animalis* CECT 8145 in moderating the glycemic response. While this finding indicates an attenuation of post-prandial glycaemic response, the absence of direct measures of insulin sensitivity (e.g., HOMA-IR or clamp-based methods) means that conclusions regarding changes in insulin sensitivity cannot be drawn from these data. Previous research in rats has shown minor alterations in the glucose and insulin response following an oral glucose tolerance test [11]. There rats were on a positive plane of nutrition and becoming obese at the time of the glucose test. Another study examining the effects of *B. animalis* CECT 8145 in dogs performed an intravenous glucose tolerance test and did not find differences in the area under the curve [14] indicating that the effect of *B. animalis* CECT 8145 on postprandial blood glucose may be mediated by a local effect within the gut. Moreover, dogs in the previous study were on a steady plane of nutrition, while dogs in the present study were in negative energy balance when a significant difference in AUC was observed. Unfortunately, the data produced during the weight gain phase of the present study was confounded by food intake. There was a large variation in how much each dog ate and how long they spent eating as they were served a full meal of 200% MER. It would be beneficial to replicate the post-prandial glucose curves during the weight gain phase using a consistent volume of food for each dog. Interestingly, the AUC and post-prandial glucose values in Phase 2 were greater in week 7 compared to week 2, after weight loss. A study performed in pregnant dairy cattle noted that animals on a low plane of nutrition, albeit short-term (48 h), also had higher glucose AUC after intravenous glucose tolerance test [17]. Fasted insulin levels increased by the end of Phase 2, which was also unexpected; however values at both time points (approximately 4 and 10 mIU/L when converted from pg/mL) were similar to previously reported fasted insulin levels for lean and healthy dogs [18,19]. These data suggest adaptation of postprandial glucose homeostasis according to calorie status. For example, the calorie deficit experienced by dogs in the weight-loss phase may have led to reduced insulin sensitivity, leading to increased postprandial blood glucose and a compensatory increase in insulin secretion. While the increase in post-prandial glucose after weight loss was unexpected, the postbiotic was able to mitigate it to an extent. Furthermore, blood glucose levels were within normal range, suggesting that dogs were not experiencing metabolic diseases like insulin resistance or diabetes.

### 4.3. Gut Hormones and Blood Chemistries

The differential effects of *B. animalis* CECT 8145 supplementation on gut hormones, particularly during the weight gain phase, provide some insights into the metabolic adaptations induced by probiotic or postbiotic supplements. In Phase 1, females receiving POST exhibited lower glucagon levels, while males showed increased GLP-1 concentrations. These findings suggest that probiotic and postbiotic supplementation may differentially affect metabolic pathways in males and females. In humans and mice, males and females exhibit differences in fat deposition and metabolism [20,21]. The study by Pedret et al. found that when separated by sex, the reduction in visceral fat mass associated with consumption of *B. animalis* CECT 8145 was only significant in females [12]. While less information is documented in dogs, it is likely that similar inherent metabolic differences between sexes exist and may have contributed to the differential group effects during weight gain. As most dogs were intact for this study and reproductive hormones play substantial roles in regulating energy metabolism, sex-influenced changes should be investigated in these types of studies. Interestingly, we observed some CBC and chemistry parameters consistently different between sexes, especially amylase and BUN, which may be reflective of differences in energy metabolism. Further investigation into sex-specific responses in energy balance and regulation of metabolism and appetite is warranted based on preliminary findings in this study, as well as how neutering/spaying impacts companion animal metabolic hormones by altering circulating levels of sex hormones. The lack of group*sex interactions in Phase 2 and inconsistent group differences when data was separated by sex could have been an artifact of reduced power after separation of the data; however innate sex differences cannot be ignored, especially regarding increasing or decreasing planes of nutrition.

Changes in blood chemistries, particularly increased GGT levels, though still within normal range, in control dogs during weight gain but not in supplemented groups, suggest potential alterations in fat metabolism or hepatic function. Previous studies have shown alterations of blood chemistry parameters in overweight animals and humans. A study by Vieira et al. (2022) evaluating the impact of overweight status (BCS of 6–7) on hematological endpoints in dogs found that overweight dogs had elevated total protein, globulin and phosphorus compared to normal weight dogs [22]. Another study by Rafaj et al. (2016) found that neutrophil count, total cholesterol concentration and alkaline phosphatase activity were increased in overweight and obese dogs [23]. In humans, elevated (yet within healthy range) levels of GGT have been associated with increased visceral fat but not subcutaneous fat [24] as well as potential marker of unfavorable body fat distribution [25]. In canines, overweight dogs have higher circulating median GGT than those with an ideal weight [26]. Unfortunately, the DEXA scans are not able to differentiate between visceral and subcutaneous fat, and there is little correlation between overall body fat percentage and the visceral to subcutaneous ratio [27]. The significant difference in GGT between CON and POST dogs observed during the weight gain phase occurred within normal veterinary reference ranges and should be interpreted cautiously, as this study did not directly assess fat distribution or hepatic function. No group effects on GGT were observed in Phase 2. While these findings indicate an association between postbiotic supplementation and circulating GGT levels during weight gain, they do not provide direct evidence of altered fat partitioning or improved metabolic or liver health.

Interestingly, several CBC and chemistry values showed inverse changes during weight loss and weight gain. While we cannot make a direct statistical comparison between the phases, our findings suggest that changes in adiposity or plane of nutrition may be reflected from standard CBC and chemistry parameters. Further research is needed to understand the implications of these changes in terms of metabolic health with weight gain or loss. The gut hormones did not reveal opposite trending changes between weight gain and weight loss phases as the CBC and chemistry data did, but changes were appropriate for each phase. Leptin increased as fat percentage increased, then decreased with loss of fat mass. During the weight loss phase, the significant decreases in GIP, glucagon, PYY, and leptin reflect the expected hormonal adaptations to reduced caloric intake. There were however some unexpected findings concerning insulin and blood glucose. Fasted glucose levels measured by the chemistry rotors revealed a decrease as dogs gained weight, though this was not observed in baseline values taken by the glucometer during the post-prandial glucose measurements which occurred on different days. The observed increase in fasting insulin alongside reduced fasting glucose during the overfeeding phase reflects a compensatory metabolic response to excess energy intake. Elevated insulin secretion facilitates glucose clearance and storage, maintaining—or even reducing—fasting glucose levels. While indicative of preserved insulin sensitivity at this stage, such a pattern may represent an early adaptive phase that precedes the development of insulin resistance with prolonged overnutrition and warrants further investigation.

Overall, supplementation did modify the levels of multiple gut hormones associated with metabolic state. One major drawback to this study was that these hormones were only assessed in a fasted state therefore we could not assess the effect of the probiotic or postbiotic on dynamic hormone secretion in response to feeding, nor directly quantify insulin sensitivity, which may differ from fasted-state measures. Post-prandial analysis in conjunction with the glucose curves would have provided a fuller picture of metabolic changes that could be happening from postbiotic supplementation. Future studies should examine how these gut hormones are also changing in response to feeding, and if supplements can cause differential post-prandial dynamics. Together, these findings support the concept that supplementation influenced metabolic regulation during periods of altered energy balance, even in the absence of measurable changes in body weight or composition.

### 4.4. Food Consumption and Digestibility

Our findings indicate that while there were no significant differences in the percentage of food consumed among groups in either phase, food intake declined steadily over time during the weight gain phase, likely reflecting a voluntary reduction in food consumption as an adaptive response to consistent intake of caloric excess leading to increased body fat. This is likely to be mediated by the increased serum leptin that paralleled weight gain. Recent studies in mice showed that *B. animalis* modulates gut microbiota derived short chain fatty acid (SCFA) production and energy metabolism in mice fed a high fat diet [28], influencing host energy metabolism via the SCFA receptor GPR43 [29]. Furthermore, the strain of *B. animalis* investigated in the present study (*B. animalis* subsp. *lactis* CECT 8145) has been shown to reduce weight gain in Zücker rats in an animal model of genetic obesity [30] and to increase lean mass and ameliorate metabolic syndrome in obese rats fed a high fat, high sugar diet [11]. Further research is warranted to investigate whether *B. animalis* CECT 8145 supplementation alters metabolic signaling in obese dogs.

Digestibility results revealed group-specific effects during both weight gain and weight loss. During weight gain, supplementation with heat-treated *B. animalis* CECT 8145 was associated with improved fat digestibility and a tendency for increased crude protein digestibility. In contrast, during the weight loss phase, supplemented groups showed trends toward lower apparent digestibility of protein and nitrogen-free extract compared with controls. These phase-dependent effects suggest that *B. animalis* CECT 8145 supplementation, particularly in its heat-treated form, may modulate nutrient digestion differently depending on energy status. As apparent total tract digestibility reflects both host nutrient absorption and microbial utilization, increased microbial metabolism during caloric restriction may manifest as reduced apparent digestibility without indicating impaired host absorption. Overall, these findings are consistent with energy status–dependent modulation of host–microbiome interactions rather than inconsistent efficacy of the postbiotic, though further research is needed to elucidate the underlying mechanisms.

### 4.5. Body Composition and Condition

As expected, significant changes in body weight and composition were observed with controlled overfeeding and calorie restriction. However, supplementation with *B. animalis* CECT 8145 did not significantly influence body weight, body condition score, or body composition during either phase of the study. While this may reflect the relatively short duration of each phase and the modest magnitude of weight change achieved, it does not necessarily indicate that the strain is ineffective for weight management. The absence of effects on body weight or composition suggests that, under the conditions of this study, the metabolic effects observed with supplementation occurred independently of changes in overall adiposity. This distinction is important, as modulation of metabolic markers may precede, or occur without, detectable changes in body mass, particularly over relatively short intervention periods. It should be noted though that dogs did not reach an extreme level of obesity or states of metabolic disease, and it is unclear if starting effects would be prominent if starting from a more obese dog population. Longer-term studies, or studies conducted in dogs with established obesity and metabolic dysfunction, would be required to determine whether these metabolic effects ultimately translate into changes in body weight or fat mass.

The observed tendency for males to have higher BCS during the weight gain phase, despite no differences in body fat percentage via DEXA scan, highlights the subjective nature of body condition scoring and underscores the importance of using objective methods like DEXA scans for more accurate assessment of body composition. As technicians handle dogs daily, small steady changes to body fat deposition were more likely to go unnoticed. A previous paper looking at using body condition scoring in managing a beagle colony noted that body condition scoring was not as sensitive in detecting weight loss as was body weight measurements, and that about 7% body weight change was needed to denote a full score change on the 5-point scale [31]. Unlike Dorsten & Cooper (2004) [31], we used a 9-point scale and did detect differences over a shorter time span, though BCS scoring may not be sensitive enough to pick up incremental changes weekly and our study did not reach the same level of body composition change.

### 4.6. Stool Quality and Fecal Markers

Supplementation with *B. animalis* CECT 8145 had minimal effects on stool quality and faecal parameters. Although statistically significant changes in stool quality scores were observed over time, these were minor and did not reflect meaningful improvements in faecal consistency, which remained on the looser end of the scale. A significant reduction in isovaleric acid was seen in Phase 1 in both the POST and PRO groups compared to controls. In contrast, Kayser et al. did not report changes in isovalerate, possibly due to differences in probiotic and postbiotic dosage or the absence of dietary changes affecting stool consistency in their study [14]. These findings are noteworthy, as short-chain fatty acids (SCFAs) play a vital role in gut health by providing energy to colonocytes and modulating immune responses. The more pronounced changes in faecal SCFA concentrations during weight gain suggest the gut microbiome may be more responsive to probiotic or postbiotic interventions when dogs are in positive energy balance. Despite alterations in SCFA and pH, no significant group effects were seen for faecal IgA or ammonia, indicating that the effects of *B. animalis* CECT 8145 on gut health may be complex and multifactorial. Interestingly, SCFA changes appeared more prominent when faecal quality was reduced. Future research should explore how these biotic interventions influence SCFA profiles in dogs with chronic diarrhoea or inflammatory bowel disease. The role of energy balance itself in shaping faecal parameters remains unclear. While most existing studies focus on macronutrient composition, such as high-fat diets, the effects of over- or underfeeding the same diet are less well characterised and warrant further investigation.

### 4.7. Limitations and Future Directions

This study has several limitations that must be considered when interpreting the data. First, all measurements were taken during supplementation, and no true baseline data were available. The absence of baseline measurements likely reduced the statistical power of the models, as inter-individual variability could not be included as a covariate; however, this was partially mitigated by inclusion of two time points per phase, allowing changes over time to be assessed. As a result, the conclusions drawn are likely to be conservative. Block randomisation was used to minimise systematic differences between groups at the start of each phase. A further limitation is that the dosage used in this study was half that employed in previous studies with the same strain [12,14,30], and stronger effects on metabolic markers may have been observed at higher doses. In addition, intra-individual comparisons between phases were not possible due to the study design: some dogs were replaced between Phase 1 and Phase 2, and dogs participating in both phases were re-randomised and may have received a different intervention. Exploratory analyses also indicated sex-specific responses to supplementation, suggesting that future studies should be designed to formally assess sex-dependent metabolic effects and associated microbiome changes.

Importantly, this study was not designed to investigate underlying mechanisms. Direct measures of insulin sensitivity, inflammatory status, microbiome composition, or tissue-specific metabolic outcomes were not assessed, limiting mechanistic interpretation of the observed changes in metabolic and fecal markers. Furthermore, dogs did not reach levels of adiposity commonly observed in household pets, likely due to study duration and the working-line genetics of the colony. As a result, metabolic changes may have been attenuated relative to those seen in chronic obesity. Finally, gut hormones were assessed only in the fasted state, which may not capture dynamic post-prandial responses. Future studies should incorporate longer intervention periods, higher doses, post-prandial hormone assessments, and mechanistic endpoints in dogs with established metabolic dysfunction to determine whether marker-level changes translate into clinically meaningful metabolic benefits.

## 5. Conclusions

In conclusion, we showed that postbiotic, and to a lesser extent probiotic, *Bifidobacterium animalis* subsp. *lactis* CECT 8145 may influence metabolic health markers without inducing short-term changes in body weight or body composition, suggesting potential metabolic health benefits that warrant further investigation. Overall, this study provides valuable insights into both the role of *B. animalis* subsp. *lactis* CECT 8145 in modulating digestive health and metabolism, as well as physiological changes in response to weight loss and weight gain in Labrador Retrievers. While the probiotic and postbiotic supplements did not significantly alter body weight or composition, they did influence gut hormone levels, nutrient digestibility, and fecal SCFA profiles, particularly during the weight gain phase. Notably, during the weight loss phase dogs in the postbiotic group had a significant reduction in postprandial glucose; this finding may prove particularly interesting for pet owners and clinicians looking for natural, non-pharmacological means of optimizing blood glucose control. These findings suggest that *B. animalis* CECT 8145 may offer specific metabolic benefits that differ according to dogs’ energy status and underscores the need for further research to fully understand its potential applications in canine health. Future studies may choose to focus on chronically obese dogs, with greater overall levels of pathology, in which larger metabolic changes may be seen. Overall, *B. animalis* subsp. *lactis* CECT 8145 appears to influence a variety of metabolic markers, providing a foundation for future research into microbiome-based interventions for canine metabolic health.

## Figures and Tables

**Table 1:** Gut hormones by experimental group and day for Phase 1 and Phase 2 of the study.

Item	Supplement						*p*-Value			
	CON		PRO		POST					
	Day 7/10	Day 45/49	Day 7/10	Day 45/49	Day 7/10	Day 45/49	Sex	Group	Time	Group * Time
**Phase 1**										
Amylin, pg/mL	1.54 ± 0.30	1.09 ± 0.31	1.49 ± 0.27	1.21 ± 0.32	1.35 ± 0.25	1.23 ± 0.38	0.96	0.99	0.16	0.79
GIP, pg/mL	2.62 ± 0.31	2.82 ± 0.57	2.78 ± 0.34	2.24 ± 0.41	2.98 ± 0.38	1.97 ± 0.33	0.16	0.81	0.033	0.078
GLP-1, pg/mL *	2.85 ± 0.69	4.05 ± 0.94	3.14 ± 0.64	5.98 ± 1.21	4.34 ± 0.96	5.44 ± 1.20	0.15	0.36	0.018	0.52
Male	2.81 ± 1.00	2.97 ± 0.86	2.84 ± 0.82	7.98 ± 2.15	6.38 ± 2.03	10.66 ± 3.10	-	0.021	0.041	0.26
Female	3.08 ± 1.03	6.04 ± 2.20	3.40 ± 0.96	4.43 ± 1.33	2.88 ± 0.88	2.48 ± 0.81	-	0.45	0.20	0.28
Ghrelin, pg/mL	288.81 ± 53.55	260.57 ± 53.55	290.16 ± 52.30	271.16 ± 51.77	320.02 ± 52.31	329.94 ± 52.86	0.78	0.75	0.48	0.66
Glucagon, pg/mL **	33.93 ± 3.38	35.01 ± 3.48	34.17 ± 3.40	33.27 ± 3.31	26.79 ± 2.67	31.22 ± 3.11	0.17	0.36	0.19	0.18
Male	28.41 ± 4.45	30.98 ± 4.86	23.43 ± 3.44	26.02 ± 3.81	31.09 ± 4.87	43.24 ± 6.78	-	0.18	0.0053	0.17
Female	40.38 ± 4.92	39.70 ± 4.84	50.29 ± 6.55	42.15 ± 5.49	22.82 ± 2.78	22.80 ± 2.78	-	0.0010	0.17	0.26
Insulin, pg/mL	94.44 ± 19.59	118.28 ± 22.34	77.49 ± 14.04	86.69 ± 14.11	128.49 ± 29.97	108.53 ± 18.59	0.46	0.31	0.48	0.20
Leptin, pg/mL *	296.07 ± 68.71	457.15 ± 105.30	208.46 ± 66.13	448.13 ± 107.22	203.14 ± 65.36	369.11 ± 105.48	0.11	0.73	<0.001	0.72
Male	105.16 ± 106.99	322.85 ± 164.10	290.53 ± 102.21	627.21 ± 164.10	142.92 ± 107.05	403.36 ± 171.87	-	0.36	0.0021	0.80
Female	483.94 ± 87.61	591.45 ± 130.97	126.39 ± 85.53	251.49 ± 134.15	260.02 ± 80.01	347.75 ± 125.07	-	0.080	0.028	0.94
PP, pg/mL	18.90 ± 2.74	16.73 ± 2.28	27.82 ± 4.89	19.27 ± 2.82	19.60 ± 2.89	18.14 ± 2.57	0.52	0.38	0.019	0.33
PYY, pg/mL	68.76 ± 14.01	64.45 ± 6.72	64.12 ± 14.51	54.02 ± 6.98	66.36 ± 14.01	55.53 ± 6.72	0.022	0.85	0.19	0.90
**Phase 2**										
Amylin, pg/mL	0.96 ± 0.74	2.84 ± 0.56	0.90 ± 0.87	2.93 ± 0.53	1.37 ± 0.66	1.80 ± 0.54	0.43	0.89	0.0036	0.21
GIP, pg/mL	3.38 ± 0.51	1.86 ± 0.27	2.20 ± 0.27	2.30 ± 0.37	2.88 ± 0.40	1.87 ± 0.27	0.94	0.86	0.0018	0.030
GLP-1, pg/mL	4.83 ± 0.64	5.64 ± 1.18	4.90 ± 0.66	6.45 ± 1.45	5.57 ± 0.80	3.67 ± 0.62	0.0051	0.49	0.94	0.064
Ghrelin, pg/mL	317.47 ± 48.22	306.73 ± 47.33	289.45 ± 48.05	285.06 ± 47.59	201.39 ± 47.33	203.07 ± 47.33	0.24	0.22	0.76	0.94
Glucagon, pg/mL	29.10 ± 5.05	27.11 ± 3.64	28.09 ± 4.86	25.49 ± 3.45	34.36 ± 5.96	24.34 ± 3.27	0.98	0.93	0.0022	0.073
Insulin, pg/mL	178.66 ± 51.24	455.34 ± 115.26	161.91 ± 51.55	359.18 ± 115.39	86.81 ± 52.46	307.34 ± 115.26	0.58	0.53	< 0.001	0.84
Leptin, pg/mL	598.23 ± 130.12	379.13 ± 106.54	489.47 ± 130.63	384.70 ± 105.19	664.11 ± 131.26	476.81 ± 105.44	0.98	0.71	< 0.001	0.46
PP, pg/mL	44.48 ± 11.72	23.42 ± 4.96	29.00 ± 12.13	31.45 ± 4.98	29.71 ± 11.72	26.57 ± 4.96	0.94	0.83	0.31	0.37
PYY, pg/mL	77.73 ± 6.46	60.36 ± 10.29	65.66 ± 7.03	60.46 ± 10.06	70.14 ± 6.39	48.19 ± 9.71	0.38	0.64	0.0037	0.35

Significant sex × group interactions: * *p* < 0.05; ** *p* < 0.01.

**Table 2:** Apparent total tract digestibility coefficients for experimental groups receiving placebo control (CON), or *B. animalis* subsp. *lactis* CECT 8145 probiotic (PRO) or heat-treated postbiotic (POST) within each phase. Differing superscripts denote significant (a and b, *p* ≤ 0.05) or trending (x and y, 0.05 < *p* ≤ 0.10) differences among experimental groups.

Phase	Item	Supplement				*p*-Value	
		CON	PRO	POST	SEM	Sex	Group
1	Dry Matter, %	91.46	91.94	92.24	0.73	0.49	0.75
	Crude Protein, %	77.93 ^y^	79.54 ^xy^	82.69 ^x^	1.48	0.30	0.072
	Crude Fat, %	91.31 ^b^	91.82 ^ab^	93.52 ^a^	0.58	0.68	0.025
	NFE, %	79.43	80.10	83.45	1.43	0.23	0.12
2	Dry Matter, %	90.12	87.01	86.76	1.45	0.077	0.16
	Crude Protein, %	76.05 ^x^	67.98 ^xy^	67.72 ^y^	2.86	0.19	0.075
	Crude Fat, %	92.48	89.52	90.23	1.03	0.094	0.12
	NFE, %	78.20 ^x^	68.18 ^y^	72.08 ^xy^	3.09	0.30	0.079

## Data Availability

The raw data supporting the conclusions of this article will be made available by the corresponding author upon reasonable request.

## Supplementary Materials

The following supporting information can be downloaded at: https://www.mdpi.com/article/10.3390/ani16020259/s1, Figure S1: Fecal quality. Figure S2: Post-prandial blood glucose for all experimental groups throughout the study in Phase 1 (A) and Phase 2 (B) showing individual datapoints.; Table S1: Average percentage of food consumed daily from food offered by experimental group for each phase. Table S2: Body composition parameters by experimental group and day. Table S3: Complete blood count results by experimental group and timepoint for Phase 1. Table S4: Serum chemistry results by experimental group and timepoint. Table S5: Fecal parameters by experimental group and day. Fecal pH and ammonia are reported from volume of fecal extract, while immunoglobulin A (IgA) and short-chain fatty acids (SCFA) are reported by gram of dry feces.

## Abbreviations

The following abbreviations are used in this manuscript:

AUC	area under the curve
BCS	Body condition score
DEXA	dual x-ray absorptiometry
GGT	Gamma-glutamyl transferase
GIP	gastric inhibitory polypeptide
GLP-1	glucagon-like peptide-1
IgA	Immunoglobulin A
PP	pancreatic polypeptide
PYY	peptide YY
SCFA	short-chain fatty acids
