# Supplementary material for: Bifidobacterium animalis Subspecies lactis CECT 8145 Affects Markers of Metabolic Health in Dogs During Weight Gain and Weight Loss
Source: Animals (Basel). 2026 Jan 15;16(2):259. doi: 10.3390/ani16020259 (PMC12837192; doi:10.3390/ani16020259)
Supplement: Supplementary file 1 [file animals-16-00259-s001.zip › animals-4034882-supplementary.pdf]

Supplementary Materials

Supplementary Table S1. Average percentage of food consumed daily from food offered by experimental group for each phase.

Phase	Item	Supplement			SEM	*P* - Value			
		CON	PRO	POST		Sex	Group	Week	Group * Week
1	Consumption, g/d	808	827	848	43	0.0091	0.81	< 0.001	0.14
	Consumption, % of offered	74.9	75.1	69.8	4.57	0.38	0.65	< 0.001	0.095
2	Consumption, g/d	559	501	557	24	0.026	0.15	< 0.001	0.30
	Consumption, % of offered	97.2	97.4	98.4	1.00	0.49	0.65	< 0.001	0.035

Supplementary Table S2, Body composition parameters by experimental group and day.

Item	Supplement						*P* - Value			
	CON		PRO		POST					
	Day 9	Day 39	Day 9	Day 39	Day 9	Day 39	Sex	Group	Time	Group * Time
Phase 1										
BMD, g/cm³	0.637 ± 0.012	0.631 ± 0.012	0.623 ± 0.012	0.616 ± 0.012	0.639 ± 0.012	0.637 ± 0.012	< 0.001	0.50	0.27	0.85
Body fat, %	17.3 ± 2.02	22.7 ± 2.46	18.4 ± 2.02	23.2 ± 2.46	18.6 ± 2.02	22.7 ± 2.46	0.24	0.96	< 0.001	0.63
Fat tissue, g	4,786 ± 614	6,604 ± 828	5,091 ± 614	6,746 ± 828	5,187 ± 614	6,751 ± 828	0.78	0.96	< 0.001	0.89
Lean tissue, g	22,907 ± 725	22,173 ± 725	22,138 ± 725	21,874 ± 725	22,815 ± 725	22,568 ± 725	< 0.001	0.78	< 0.001	0.098
Total tissue mass, g	27,689 ± 738	28,773 ± 860	27,233 ± 738	28,624 ± 860	27,997 ± 738	29,250 ± 860	< 0.001	0.82	< 0.001	0.83
Lean:Fat ratio	5.72 ± 1.09	4.02 ± 0.80	5.49 ± 1.09	4.09 ± 0.80	6.63 ± 1.09	5.01 ± 0.80	0.52	0.68	< 0.001	0.89
Phase 2										
BMD, g/cm³	0.632 ± 0.013	0.635 ± 0.013	0.663 ± 0.013	0.665 ± 0.013	0.626 ± 0.013	0.648 ± 0.013	< 0.001	0.14	0.12	0.32
Body fat, %	31.8 ± 2.30	27.7 ± 2.73	30.7 ± 2.32	27.5 ± 2.74	31.9 ± 2.30	29.8 ± 2.73	0.61	0.88	< 0.001	0.63
Fat tissue, g	9,710 ± 842	8,208 ± 933	9,594 ± 847	8,142 ± 937	9,901 ± 842	8,673 ± 933	0.15	0.94	< 0.001	0.88
Lean tissue, g	20,790 ± 744	21,035 ± 744	21,304 ± 749	20,936 ± 749	20,833 ± 744	20,286 ± 744	< 0.001	0.86	0.29	0.27
Total tissue, g	30,497 ± 799	29,241 ± 925	30,889 ± 803	29,069 ± 929	30,731 ± 799	28,287 ± 925	< 0.001	0.92	< 0.001	0.27
Lean:Fat ratio	2.39 ± 0.31	3.23 ± 0.68	2.50 ± 0.31	3.59 ± 0.68	2.53 ± 0.31	3.11 ± 0.68	0.46	0.92	0.0075	0.79

Abbreviations: BMD, bone mineral density.

Classification: Confidential

Supplementary Table S3. Complete blood count results by experimental group and timepoint for Phase 1.

	Supplement						*P* - Value			
	CON		PRO		POST					
	Day 7/10	Day 45/49	Day 7/10	Day 45/49	Day 7/10	Day 45/49	Sex	Group	Time	Group * Time
**Phase 1**										
WBC, 10^9/L	10.64 ± 0.61	10.47 ± 0.59	9.30 ± 0.47	10.16 ± 0.56	10.51 ± 0.60	10.17 ± 0.56	0.17	0.48	0.58	0.12
Lym, 10^9/L	1.73 ± 0.16	1.55 ± 0.12	1.37 ± 0.16	1.61 ± 0.12	1.72 ± 0.16	1.65 ± 0.12	0.61	0.49	0.95	0.21
Mon, 10^9/L	0.48 ± 0.06	0.44 ± 0.04	0.43 ± 0.05	0.39 ± 0.04	0.44 ± 0.05	0.44 ± 0.04	0.0044	0.64	0.46	0.80
Neu, 10^9/L	8.31 ± 0.52	8.28 ± 0.51	7.29 ± 0.40	7.96 ± 0.47	8.22 ± 0.51	7.87 ± 0.46	0.11	0.52	0.60	0.14
Eos, 10^9/L	0.11 ± 0.03	0.15 ± 0.03	0.12 ± 0.03	0.15 ± 0.03	0.12 ± 0.03	0.11 ± 0.03	0.14	0.81	0.026	0.18
Bas, 10^9/L	0.04 ± 0.01	0.05 ± 0.01	0.03 ± 0.01	0.05 ± 0.01	0.03 ± 0.01	0.04 ± 0.01	0.092	0.54	< 0.001	0.22
RBC, 10^12/L	7.17 ± 0.14	7.27 ± 0.14	7.09 ± 0.14	7.39 ± 0.14	6.85 ± 0.14	7.20 ± 0.14	0.35	0.42	0.0010	0.30
HGB, g/dL	15.24 ± 0.28	15.44 ± 0.28	14.93 ± 0.28	15.36 ± 0.28	14.73 ± 0.28	15.20 ± 0.28	0.61	0.56	0.017	0.73
HCT, %	46.65 ± 0.83	48.19 ± 0.83	45.52 ± 0.83	47.78 ± 0.83	44.22 ± 0.83	47.29 ± 0.83	0.095	0.27	< 0.001	0.44
MCV, fl	64.77 ± 0.61	66.31 ± 0.61	64.29 ± 0.61	64.69 ± 0.61	64.57 ± 0.61	65.84 ± 0.61	0.22	0.43	< 0.001	0.11
MCH, pg	21.29 ± 0.19	21.27 ± 0.19	21.05 ± 0.19	20.81 ± 0.19	21.49 ± 0.19	21.12 ± 0.19	0.32	0.27	0.011	0.18
MCHC, g/dL	32.81 ± 0.23	32.04 ± 0.23	32.77 ± 0.23	32.15 ± 0.23	33.30 ± 0.23	32.17 ± 0.23	0.0031	0.48	< 0.001	0.26
RDWc, %	16.83 ± 0.16	17.20 ± 0.16	16.90 ± 0.16	17.24 ± 0.16	16.63 ± 0.16	17.16 ± 0.16	0.39	0.61	< 0.001	0.78
RDWs, fl	40.90 ± 0.36	42.88 ± 0.36	40.76 ± 0.36	42.29 ± 0.36	40.38 ± 0.36	42.84 ± 0.36	0.0011	0.64	< 0.001	0.34
PLT, 10^9/L	240.94 ± 14.85	265.08 ± 14.85	210.18 ± 14.85	228.59 ± 14.85	218.48 ± 14.85	244.74 ± 14.85	0.26	0.21	0.0045	0.91
MPV, fl	8.94 ± 0.22	8.63 ± 0.22	8.74 ± 0.22	8.80 ± 0.22	9.23 ± 0.22	8.81 ± 0.22	0.91	0.57	0.077	0.26
PCT, %	0.22 ± 0.02	0.23 ± 0.01	0.17 ± 0.02	0.20 ± 0.01	0.20 ± 0.02	0.21 ± 0.01	0.21	0.12	0.072	0.50
PDWc, %	35.83 ± 0.55	35.45 ± 0.55	36.14 ± 0.55	35.61 ± 0.55	36.72 ± 0.55	36.40 ± 0.55	0.89	0.41	0.13	0.95
PDWs, fl	13.00 ± 0.51	12.40 ± 0.59	13.26 ± 0.54	12.78 ± 0.63	14.17 ± 0.61	13.30 ± 0.68	0.52	0.41	0.030	0.91
**Phase 2**										
WBC, 10^9/L	8.90 ± 0.52	8.34 ± 0.52	8.06 ± 0.52	7.63 ± 0.52	8.07 ± 0.52	8.46 ± 0.52	0.31	0.46	0.56	0.45
Lym, 10^9/L	1.49 ± 0.12	1.34 ± 0.12	1.48 ± 0.12	1.38 ± 0.12	1.38 ± 0.12	1.57 ± 0.12	0.37	0.90	0.81	0.17

Classification: Confidential

Mon, 10^9/L	0.55 ± 0.11	0.33 ± 0.04	0.37 ± 0.11	0.35 ± 0.04	0.37 ± 0.11	0.40 ± 0.04	0.88	0.68	0.22	0.21
Neu, 10^9/L	6.81 ± 0.46	6.55 ± 0.46	6.08 ± 0.46	5.81 ± 0.46	6.04 ± 0.46	6.37 ± 0.46	0.34	0.40	0.82	0.65
Eos, 10^9/L	0.12 ± 0.03	0.07 ± 0.02	0.08 ± 0.02	0.06 ± 0.01	0.09 ± 0.02	0.08 ± 0.02	0.54	0.65	0.032	0.33
Bas, 10^9/L	0.04 ± 0.008	0.03 ± 0.007	0.03 ± 0.007	0.02 ± 0.006	0.03 ± 0.007	0.03 ± 0.006	0.96	0.55	0.0077	0.43
RBC, 10^12/L	7.23 ± 0.13	7.00 ± 0.13	7.20 ± 0.13	7.15 ± 0.13	7.25 ± 0.13	7.10 ± 0.13	0.66	0.92	0.015	0.43
HGB, g/dL	15.25 ± 0.28	14.67 ± 0.28	14.92 ± 0.29	14.92 ± 0.29	15.45 ± 0.28	14.96 ± 0.28	0.48	0.70	0.012	0.17
HCT, %	47.98 ± 0.77	45.51 ± 0.77	47.32 ± 0.78	45.90 ± 0.78	48.34 ± 0.77	46.34 ± 0.77	0.75	0.73	< 0.001	0.51
MCV, fl	66.51 ± 0.63	65.25 ± 0.48	65.47 ± 0.63	64.34 ± 0.48	66.78 ± 0.63	65.31 ± 0.48	0.25	0.21	< 0.001	0.91
MCH, pg	21.11 ± 0.19	20.99 ± 0.26	20.72 ± 0.19	20.90 ± 0.26	21.31 ± 0.19	21.09 ± 0.26	0.42	0.37	0.69	0.47
MCHC, g/dL	31.76 ± 0.29	32.20 ± 0.29	31.64 ± 0.29	32.53 ± 0.29	31.94 ± 0.29	32.28 ± 0.29	0.18	0.90	0.024	0.61
RDWc, %	17.32 ± 0.16	16.80 ± 0.16	17.52 ± 0.16	17.13 ± 0.16	17.27 ± 0.16	16.87 ± 0.16	0.28	0.31	< 0.001	0.84
RDWs, fl	43.69 ± 0.43	40.74 ± 0.43	43.41 ± 0.43	40.89 ± 0.43	43.69 ± 0.43	41.17 ± 0.43	0.033	0.80	< 0.001	0.85
PLT, 10^9/L	270.99 ± 19.50	273.64 ± 19.69	265.50 ± 19.19	247.97 ± 17.93	240.01 ± 17.27	211.45 ± 15.22	0.43	0.11	0.13	0.39
MPV, fl	8.88 ± 0.19	9.78 ± 0.19	8.49 ± 0.19	9.68 ± 0.19	8.82 ± 0.19	9.57 ± 0.19	0.44	0.54	< 0.001	0.34
PCT, %	0.24 ± 0.02	0.27 ± 0.02	0.22 ± 0.02	0.24 ± 0.02	0.21 ± 0.02	0.20 ± 0.01	0.30	0.079	0.31	0.40
PDWc, %	35.75 ± 0.40	37.78 ± 0.40	34.66 ± 0.40	37.62 ± 0.40	35.55 ± 0.40	37.59 ± 0.40	0.52	0.38	< 0.001	0.31
PDWs, fl	13.23 ± 0.52	15.71 ± 0.52	12.16 ± 0.52	15.27 ± 0.52	12.89 ± 0.52	15.49 ± 0.52	0.35	0.46	< 0.001	0.71

Significant sex*group interactions: *P < 0.05; **P < 0.01. Abbreviations: WBC, white blood cells; Lym, lymphocytes; Mon, monocytes; Neu, neutrophils; Eos, eosinophils; Bas, basophils; RBC, red blood cells; HGB, hemoglobin; HCT, hematocrit; MCV, mean corpuscular volume; MCH, mean corpuscular hemoglobin; MCHC, mean corpuscular hemoglobin concentration; RDW, red blood cell distribution; RDWs, RDW standard deviation; RDWc, RDW coefficient of variation; PLT, platelets; MPV, mean platelet volume; PCT, plateletcrit; PDW, platelet distribution width; PDWc PDW coefficient of variation; PDWs, PDW standard deviation.

Supplementary Table S4. Serum chemistry results by experimental group and timepoint.

	Supplement						*P* - Value			
	**CON**		**PRO**		**POST**					
	**Day 7/10**	**Day 45/49**	**Day 7/10**	**Day 45/49**	**Day 7/10**	**Day 45/49**	**Sex**	**Group**	**Time**	**Group * Time**

**Phase 1**										
ALB, g/dL	3.81 ± 0.05	3.87 ± 0.05	3.79 ± 0.05	3.84 ± 0.05	3.64 ± 0.05	3.78 ± 0.05	0.0037	0.15	< 0.001	0.22
ALP, U/L	49.06 ± 4.69	49.91 ± 4.77	46.18 ± 4.41	51.88 ± 4.95	50.74 ± 4.85	47.68 ± 4.55	0.015	0.99	0.46	0.084
ALT, U/L	45.53 ± 3.62	43.07 ± 3.62	53.70 ± 3.62	50.97 ± 3.62	49.30 ± 3.62	44.93 ± 3.62	0.45	0.24	0.055	0.87
AMY, U/L	666.63 ± 45.25	721.97 ± 45.25	671.03 ± 45.25	719.57 ± 45.25	631.57 ± 45.25	664.23 ± 45.25	0.016	0.69	0.0012	0.77
TBIL, mg/dL	0.26 ± 0.01	0.27 ± 0.01	0.27 ± 0.01	0.29 ± 0.01	0.26 ± 0.01	0.29 ± 0.01	0.019	0.30	< 0.001	0.32
BUN, mg/dL	16.27 ± 1.09	16.57 ± 1.09	16.93 ± 1.09	17.43 ± 1.09	16.77 ± 1.09	16.84 ± 1.09	0.012	0.87	0.56	0.94
Ca, mg/dL	10.22 ± 0.08	10.28 ± 0.08	10.34 ± 0.08	10.44 ± 0.08	10.21 ± 0.08	10.38 ± 0.08	0.26	0.39	0.025	0.59
Phos, mg/dL	4.88 ± 0.15	5.10 ± 0.15	4.90 ± 0.15	5.01 ± 0.15	4.97 ± 0.15	4.79 ± 0.15	0.37	0.78	0.63	0.28
Cre, mg/dL	0.82 ± 0.04	0.89 ± 0.06	0.87 ± 0.04	0.83 ± 0.05	0.80 ± 0.04	0.91 ± 0.06	0.12	0.99	0.12	0.078
Glu, mg/dL	101.69 ± 1.65	96.75 ± 1.65	100.25 ± 1.65	94.65 ± 1.65	99.29 ± 1.65	91.35 ± 1.65	0.12	0.16	< 0.001	0.44
Na+, nmol/L	148.82 ± 0.56	146.69 ± 0.56	147.38 ± 0.56	146.78 ± 0.56	147.62 ± 0.56	147.09 ± 0.56	0.22	0.56	0.0084	0.18
K+, nmol/L	4.73 ± 0.06	4.75 ± 0.06	4.69 ± 0.06	4.69 ± 0.06	4.61 ± 0.06	4.53 ± 0.06	0.82	0.076	0.56	0.54
TP, g/dL	5.92 ± 0.08	5.93 ± 0.08	5.99 ± 0.08	6.00 ± 0.08	5.89 ± 0.08	5.99 ± 0.08	0.56	0.75	0.29	0.46
Glob, g/dL	2.02 ± 0.07	2.05 ± 0.07	2.17 ± 0.07	2.12 ± 0.07	2.18 ± 0.07	2.18 ± 0.07	0.11	0.30	0.75	0.49
GGT, U/L	4.30 ± 0.35	3.90 ± 0.22	3.36 ± 0.35	3.70 ± 0.22	3.10 ± 0.35	3.24 ± 0.22	0.0014	0.050	0.88	0.13
BA, µmol/L †	0 ± 0	0 ± 0	1.9 ± 1.2	0 ± 0	0 ± 0	0 ± 0	N/A	N/A	N/A	N/A
Chol, mg/dL	204.25 ± 12.50	224.99 ± 12.50	212.41 ± 12.50	226.68 ± 12.50	199.99 ± 12.50	210.19 ± 12.50	0.022	0.68	0.0029	0.66
TG, mg/dL	44.22 ± 2.56	45.36 ± 2.56	48.04 ± 2.56	49.64 ± 2.56	46.76 ± 2.56	44.89 ± 2.56	0.58	0.33	0.88	0.72
**Phase 2**										
ALB, g/dL	3.28 ± 0.08	3.15 ± 0.08	3.12 ± 0.08	3.04 ± 0.08	3.31 ± 0.08	3.23 ± 0.08	0.23	0.12	0.067	0.89
ALP, U/L	52.61 ± 5.45	45.01 ± 5.45	43.19 ± 5.49	33.93 ± 5.49	52.24 ± 5.45	44.84 ± 5.45	0.55	0.31	< 0.001	0.86
ALT, U/L	45.70 ± 4.59	47.70 ± 5.94	46.94 ± 4.62	49.64 ± 5.96	53.70 ± 4.59	52.70 ± 5.94	0.45	0.59	0.68	0.87
AMY, U/L	704.32 ± 44.60	699.66 ± 44.60	682.44 ± 44.85	640.24 ± 44.85	653.52 ± 44.60	604.79 ± 44.60	< 0.001	0.49	0.022	0.36
TBIL, mg/dL	0.34 ± 0.01	0.34 ± 0.01	0.33 ± 0.01	0.33 ± 0.01	0.36 ± 0.01	0.35 ± 0.01	< 0.001	0.24	1.0	0.84
BUN, mg/dL	14.33 ± 0.77	15.76 ± 0.77	12.12 ± 0.78	14.56 ± 0.78	13.60 ± 0.77	15.00 ± 0.77	< 0.001	0.26	< 0.001	0.29

Classification: Confidential

Ca, mg/dL	10.44 ± 0.09	10.38 ± 0.09	10.26 ± 0.09	10.10 ± 0.09	10.55 ± 0.09	10.44 ± 0.09	0.75	0.0076	0.065	0.80
Phos, mg/dL	4.65 ± 0.11	4.72 ± 0.11	4.58 ± 0.11	4.64 ± 0.11	4.71 ± 0.11	4.64 ± 0.11	0.031	0.82	0.78	0.69
Cre, mg/dL	0.93 ± 0.06	1.04 ± 0.06	0.85 ± 0.06	0.95 ± 0.06	0.84 ± 0.06	0.90 ± 0.06	0.056	0.25	< 0.001	0.72
Glu, mg/dL	91.86 ± 2.04	90.86 ± 1.54	92.52 ± 2.05	93.85 ± 1.55	92.86 ± 2.04	92.40 ± 1.54	0.0012	0.72	0.96	0.57
Na+, nmol/L	146.88 ± 0.51	145.08 ± 0.51	146.45 ± 0.51	144.65 ± 0.51	147.35 ± 0.51	145.62 ± 0.51	0.0047	0.30	< 0.001	1.0
K+, nmol/L	4.63 ± 0.07	4.62 ± 0.07	4.64 ± 0.07	4.63 ± 0.07	4.62 ± 0.07	4.64 ± 0.07	0.14	1.0	1.0	0.98
TP, g/dL	6.36 ± 0.09	6.25 ± 0.07	6.28 ± 0.09	6.14 ± 0.07	6.25 ± 0.09	6.13 ± 0.07	0.21	0.50	< 0.001	0.95
Glob, g/dL	3.11 ± 0.12	3.27 ± 0.08	3.19 ± 0.12	3.23 ± 0.08	2.95 ± 0.12	3.11 ± 0.08	0.68	0.32	0.034	0.59
GGT, U/L	3.64 ± 0.24	3.38 ± 0.24	3.66 ± 0.24	3.66 ± 0.24	3.98 ± 0.24	3.98 ± 0.24	0.0081	0.30	0.50	0.64
BA, μmol/L †	0.07 ± 0.31	0.00 ± 0.14	0.00 ± 0.31	0.34 ± 0.14	0.53 ± 0.31	0.00 ± 0.14	0.91	0.63	0.65	0.21
Chol, mg/dL	239.70 ± 11.54	227.50 ± 11.54	208.62 ± 11.61	204.09 ± 11.61	211.10 ± 11.54	200.96 ± 11.54	0.079	0.14	0.027	0.71
TG, mg/dL	51.14 ± 4.51	53.60 ± 4.51	46.55 ± 4.53	49.41 ± 4.53	48.14 ± 4.51	44.54 ± 4.51	0.39	0.55	0.81	0.45

†model for bile acid data did not converge, thus raw data means and SEMs are presented. Most all values reported from the rotors were 0 at each timepoint. Abbreviations: ALB, albumin; ALP, alkaline phosphatase; ALT, alanine aminotransferase; AMY, amylase; TBIL, total bilirubin; BUN, blood urea nitrogen; Ca, calcium; Phos, phosphorus; Cre, creatinine; Glu, glucose; Na+, sodium; K+, potassium; TP, total protein; Glob, globulin; GGT, gamma-glutamyl transferase; BA, bile acids; Chol, cholesterol; TG, triglycerides.

Supplementary Table S5. Fecal parameters by experimental group and day. Fecal pH and ammonia are reported from volume of fecal extract, while immunoglobulin A (IgA) and short-chain fatty acids (SCFA) are reported by gram of dry feces.

**Item**	**Supplement**						***P* - Value**			
	**CON**		**PRO**		**POST**					
	**Day 9**	**Day 39**	**Day 9**	**Day 39**	**Day 9**	**Day 39**	**Sex**	**Group**	**Time**	**Group * Time**
				Phase 1						
Moisture, %	72.82 ± 0.77	72.24 ± 0.77	73.73 ± 0.77	70.34 ± 0.77	71.54 ± 0.77	71.15 ± 0.77	0.24	0.24	0.041	0.15
pH	6.45 ± 0.09	6.25 ± 0.09	6.36 ± 0.09	6.22 ± 0.09	6.19 ± 0.09	6.38 ± 0.09	0.26	0.76	0.45	0.046
IgA, mg/g	7.11 ± 1.34	2.74 ± 0.67	5.40 ± 1.34	1.40 ± 0.67	4.83 ± 1.34	2.46 ± 0.67	0.21	0.29	< 0.001	0.62
Ammonia, g/L	1.66 ± 0.12	1.33 ± 0.12	1.44 ± 0.12	1.38 ± 0.12	1.23 ± 0.12	1.49 ± 0.12	0.076	0.80	0.83	0.073

Classification: Confidential

Acetate, μmol/g	412.48 ± 19.61	431.85 ± 29.87	379.44 ± 19.61	513.25 ± 29.87	391.07 ± 19.61	422.70 ± 29.87	0.62	0.35	0.0022	0.034
Propionate, μmol/g	191.91± 21.40	182.62 ± 21.40	187.80 ± 21.40	254.46 ± 21.40	204.23 ± 21.40	174.61 ± 21.40	0.69	0.24	0.59	0.062
Isobutyrate, μmol/g	8.06 ± 0.49	6.92 ± 0.49	5.51 ± 0.49	7.17 ± 0.49	5.71 ± 0.49	6.90 ± 0.49	0.40	0.038	0.14	0.010
Butyrate, μmol/g	90.01 ± 6.06	78.06 ± 6.06	70.72 ± 6.06	88.29 ± 6.06	70.54 ± 6.06	91.85 ± 6.06	0.81	0.76	0.069	0.014
Isovalerate, μmol/g	10.45 ± 0.59	9.24 ± 0.59	7.76 ± 0.59	9.30 ± 0.59	7.27 ± 0.59	8.92 ± 0.59	0.99	0.018	0.16	0.025
Valerate, μmol/g	1.19 ± 0.33	0.65 ± 0.13	0.63 ± 0.18	1.16 ± 0.24	1.13 ± 0.32	0.66 ± 0.13	0.22	0.99	0.38	0.028
Phase 2										
Moisture, %	69.16 ± 1.10	69.75 ± 1.10	70.82 ± 1.10	69.34 ± 1.10	69.67 ± 1.10	69.88 ± 1.10	0.13	0.86	0.79	0.58
pH	6.30 ± 0.08	6.14 ± 0.08	6.37 ± 0.08	6.25 ± 0.08	6.41 ± 0.08	6.15 ± 0.08	0.015	0.56	0.012	0.69
IgA, mg/g	8.77 ± 3.14	1.36 ± 0.43	2.33 ± 3.14	0.81 ± 0.44	4.46 ± 3.14	1.21 ± 0.43	0.19	0.29	0.034	0.42
Ammonia, g/L	0.91 ± 0.09	1.14 ± 0.08	0.72 ± 0.08	1.04 ± 0.07	0.85 ± 0.09	1.08 ± 0.07	0.25	0.21	< 0.001	0.66
Acetate, μmol/g	394.22 ± 25.16	433.85 ± 25.16	411.28 ± 25.26	370.00 ± 25.26	378.47 ± 25.16	389.66 ± 25.16	0.23	0.56	0.85	0.15
Propionate, μmol/g	227.49 ± 15.92	241.49 ± 15.92	239.16 ± 15.97	222.66 ± 15.97	183.61 ± 15.92	212.86 ± 15.92	< 0.001	0.062	0.48	0.33
Isobutyrate, μmol/g	8.44 ± 0.63	7.52 ± 0.63	7.04 ± 0.64	7.26 ± 0.64	6.79 ± 0.63	7.46 ± 0.63	0.96	0.39	0.98	0.35
Butyrate, μmol/g	88.11 ± 6.34	78.44 ± 6.34	70.93 ± 6.36	65.30 ± 6.36	78.42 ± 6.34	68.26 ± 6.34	0.094	0.11	0.067	0.90
Isovalerate, μmol/g	9.85 ± 0.76	8.96 ± 0.73	8.09 ± 0.70	7.67 ± 0.68	8.12 ± 0.69	7.81 ± 0.68	0.58	0.095	0.33	0.92
Valerate, μmol/g	5.59 ± 2.00	2.78 ± 1.14	1.95 ± 2.01	2.33 ± 1.15	1.83 ± 2.00	2.68 ± 1.14	0.80	0.46	0.65	0.38

Classification: Confidential

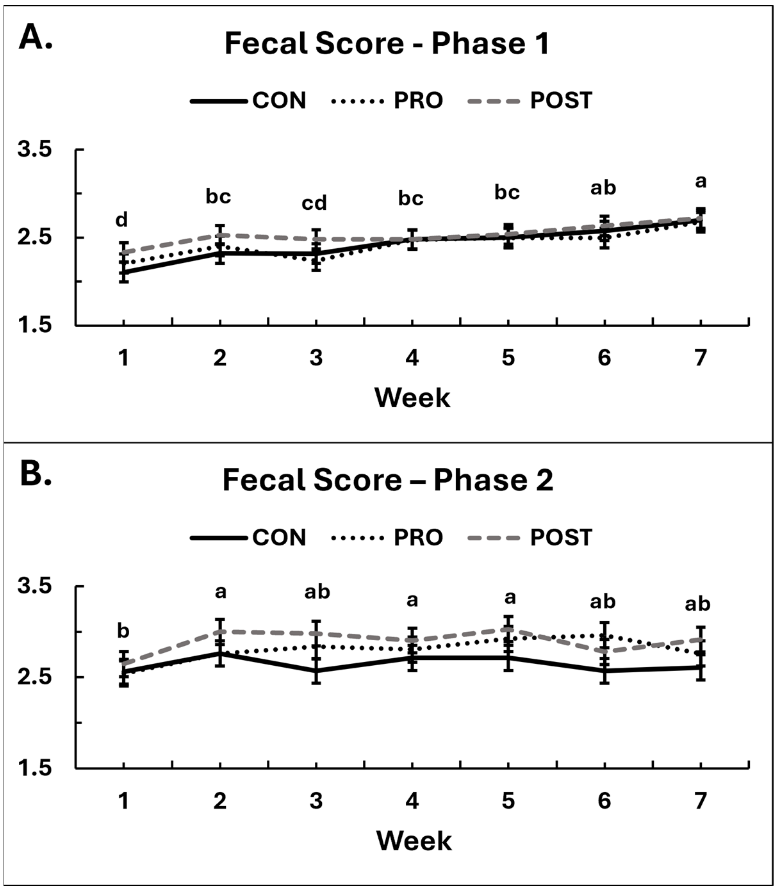

Supplementary Figure S1. Fecal quality. Average weekly fecal scores for all experimental groups throughout the study in Phase 1 (A) and Phase 2 (B). Differing superscripts denote significant differences by week ($P < 0.05$).

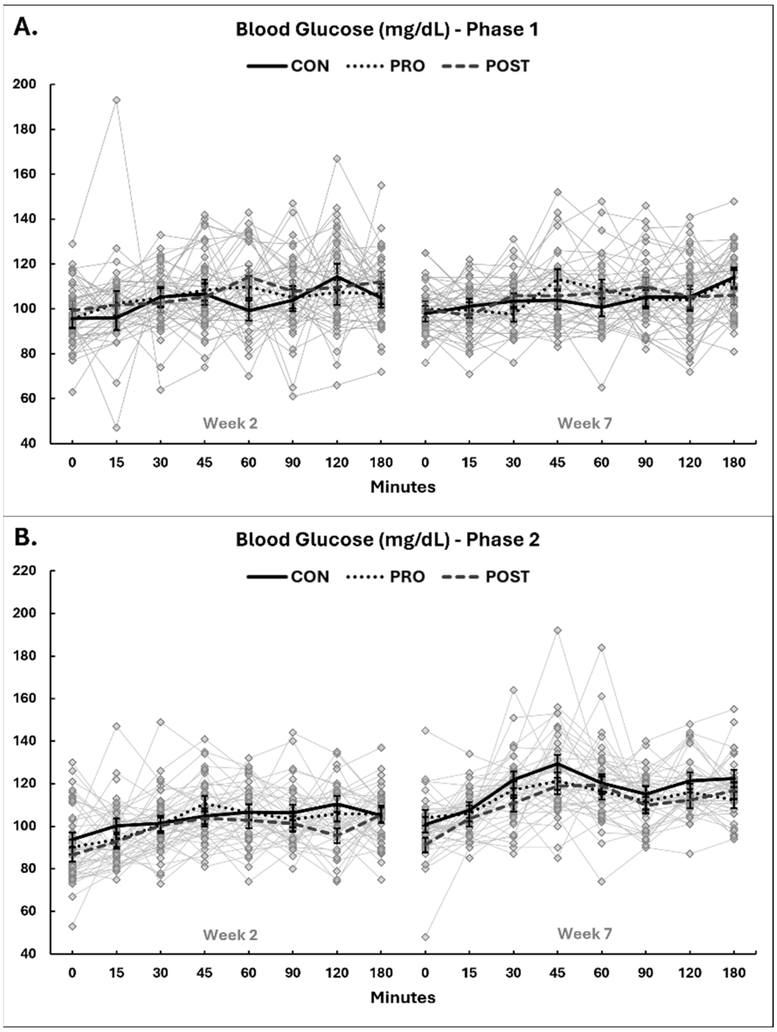

Supplementary Figure S2. Post-prandial blood glucose for all experimental groups throughout the study in Phase 1 (A) and Phase 2 (B) showing individual datapoints. Time 0 represents baseline glucose levels before the meal. Means sharing the same superscript letter are not significantly different; means with different superscripts differ significantly between weeks ($P < 0.05$).
